# Coral restoration: roles of shelter for herbivores and reef state in early recruitment success

**DOI:** 10.7717/peerj.20891

**Published:** 2026-04-07

**Authors:** Eric R. Dilley, Erik G. Brush, Ryan N. Jones, Mark A. Hixon

**Affiliations:** 1 School of Life Sciences, University of Hawaiʻi at Mānoa, Honolulu, Hawaiʻi, United States of America; 2 State of Hawaiʻi Division of Aquatic Resources, Honolulu, Hawaiʻi, United States of America

**Keywords:** Juvenile coral, Demography, Recruitment, Survival, Growth, Hawaiʻi, Herbivory, Reefscape, Shelter

## Abstract

Coral recruitment is crucial for reef recovery, yet our knowledge of recruit and juvenile coral demographics remains limited. To experimentally test whether shelter for herbivores and a healthy reefscape enhance colonization by corals, we deployed cubic-meter concrete modules in both low- and high-shelter configurations at the degraded reef off Waikı¯kı¯ Beach and the relatively healthy and fully protected reef at Hanauma Bay, Oʻahu, Hawaiʻi. Settling corals of three dominant genera (*Pocillopora*, *Montipora*, and *Porites*) were surveyed quarterly to track individual recruitment, survival, and growth for nearly four years. There was greater colonization of modules by sea urchins, higher visitation by herbivorous fishes, and lower algal overgrowth of recruit and juvenile corals at Hanauma Bay compared to Waikı¯kı¯. Nonetheless, demographic responses by corals were often genus, site, and treatment specific, and failed to strongly corroborate three *a priori* hypotheses. The hypothesis that herbivores enhance recruit and juvenile coral demographic metrics was partially evident for survival and growth of *Montipora*, as well as survival of *Porites* which were positively correlated with herbivorous fish biomass. Additionally, *Pocillopora* recruitment and survival were negatively correlated with algal overgrowth. The related hypothesis that increased shelter for herbivores indirectly benefits corals was also mostly rejected, except for recruitment of *Pocillopora* at Hanauma Bay and for survival of *Pocillopora* and *Porites*. A third hypothesis that a healthy reefscape benefits corals was corroborated for *Pocillopora* survival and *Montipora* recruitment and growth, yet not for *Porites*. Despite the relatively low number of correlations associated with our three *a priori* hypotheses, results from this study still suggest that recruit and juvenile corals are most likely to thrive in reef environments with sufficient herbivory and lower nutrients that fertilize algal growth. We conclude that further place-based management of herbivores is needed to replenish herbivore populations, increase the effectiveness of coral restoration efforts, and enhance natural coral recovery on Hawaiian reefs.

## Introduction

Coral reefs are hotspots of marine biodiversity that provide a broad variety of ecosystem goods and services for society (Woodhead et al., 2019). However, these ecosystem benefits are at risk due to climate disruption and other human disturbances that reduce ecological resilience (Eddy et al., 2021) and facilitate coral reef decline worldwide. Understanding the processes that foster coral resilience is therefore crucial for predicting how corals will fare in a changing climate and how reefs can be managed and restored to ensure their persistence. Effective coral restoration efforts will rely on understanding the factors enhancing or inhibiting recruit (≤10 mm) and juvenile corals (≤50 mm: Doropoulos et al., 2015) that replenish reefs, knowledge which requires long-term field studies with high sampling frequency. Although valuable, prior studies of recruit and juvenile coral dynamics have surveyed colonies either once per year over long periods (*e.g.*, Hughes, 1996; Connell, Hughes & Wallace, 1997) or multiple times per year over short periods (*e.g.*, Oren & Benayahu, 1997; Martinez & Abelson, 2013) rather than both.

Coral populations are limited by factors affecting the rate of larval settlement and recruitment, colony survival, and subsequent growth (Ritson-Williams et al., 2009; Arnold & Steneck, 2011). Besides physical disturbances, such as major storms and coral bleaching events, small-scale biotic interactions such as predation, incidental damage (ammensalism), and competition for space fundamentally affect corals. Predation and ammensalism are important ecological interactions that negatively affect coral recruits and juveniles. Corallivorous fishes (Rotjan & Lewis, 2008; Penin et al., 2011) and invertebrates (Rotjan & Lewis, 2008) can substantially reduce survival rates of juvenile corals on exposed surfaces, yet predation is reduced in microhabitats such as crevices that protect small recruits (Doropoulos et al., 2016). Additionally, ammensalism by excavating fishes (Penin et al., 2010; Lenihan et al., 2011) and urchins (O’Leary et al., 2013) is known to locally reduce coral recruitment by incidental scraping or chipping of coral skeletons causing partial or complete colony mortality.

Corals also face intense competition for space during settlement and recruitment to the benthos. Macroalgae and turf algae are effective space competitors that can impede coral recruits and juveniles (Arnold, Steneck & Mumby, 2010; Ceccarelli et al., 2018). These algae can reduce coral settlement by preempting favorable settlement microhabitats of coral planulae (Doropoulos et al., 2016). Some algae also secrete harmful allelochemicals that can inhibit coral settlement (Kuffner et al., 2006; Evensen et al., 2019; Vizon et al., 2024). Small juvenile corals are susceptible to direct algal overgrowth (Birrell et al., 2008; Arnold, Steneck & Mumby, 2010) and can be damaged by mechanical abrasion from algal thalli (Box & Mumby, 2007), both of which can reduce growth (Venera-Ponton et al., 2011) or cause mortality (Penin et al., 2011; Doropoulos et al., 2016). Additionally, algal turfs can harbor high concentrations of microbes that cause coral disease (Smith et al., 2006).

Benthic algae grow faster with elevated nutrients (Smith, Smith & Hunter, 2001), which are frequently associated with sewage discharge and both agricultural and urban runoff, resulting in more frequent algal overgrowth of corals (Vermeij et al., 2010). Reefs with greater nutrient inputs are also known to have reduced coral recruitment (Hunte & Wittenberg, 1992) and survival (Wittenberg & Hunte, 1992). Areas with higher nutrient levels are also associated with more abundant colonial sponge and ascidian communities (Ward-Paige et al., 2005; Shenkar, Bronstein & Loya, 2008) that can prevent coral planulae from settling or overgrow recruit and juvenile corals (Arnold & Steneck, 2011; Brandt et al., 2019).

The negative effects of macroalgae and turf algae can be mitigated by abundant and diverse assemblages of herbivorous fishes and invertebrates, which crop algae and reduce spatial competition with corals (Mumby & Steneck, 2008; Hixon, 2015; Williams et al., 2019). Herbivory has also been shown to reduce algal abundance to the benefit of corals in Hawaiʻi (Smith, Smith & Hunter, 2001; Gove et al., 2023). More specifically, herbivory by fishes opens living space for settlement of coral larvae, thereby increasing the potential for coral recruitment (Mumby, 2009; Arnold, Steneck & Mumby, 2010), survival (Birkeland, 1977), and growth (Suchley & Alvarez-Filip, 2017). Sea urchin grazing is also known to reduce coral-algal competition, resulting in increased recruitment (Carpenter & Edmunds, 2006), survival (Idjadi, Haring & Precht, 2010), and growth (Davies & Vize, 2008; Idjadi, Haring & Precht, 2010) of juvenile corals. Additionally urchin grazing can dislodge small sponges that could otherwise overgrow corals (Vance, 1979). Human interventions to remove dense macroalgae can increase coral recruitment as well (Smith et al., 2022).

Healthy reefscapes are generally characterized by less macroalgae capable of overgrowing corals, greater abundance of structurally complex habitat, and higher rates of coral recruitment, particularly within marine reserves where herbivorous fishes (Mumby et al., 2007; Steneck et al., 2018) and coral larvae (Vermeij & Sandin, 2008; Da-Anoy et al., 2017) are typically more abundant. Some species of crustose coralline algae, typical on highly grazed, protected reefs, are known to enhance coral larval settlement (Ritson-Williams et al., 2009).

On highly degraded reefs, dead coral colonies eventually erode to low-relief rubble (Alvarez-Filip et al., 2009). The resulting lack of physical shelter reduces the abundance of herbivores, such that collapsed reefs frequently become dominated by algal turfs and macroalgae (Mumby & Steneck, 2008; Hixon, 2015). Herbivore abundance is enhanced by the availability of structural shelter in habitats where they forage, serving as nearby refugia from predation and extreme water motion (Graham & Nash, 2013; Madin et al., 2019). Therefore, artificial structures can serve as coral restoration tools by immediately increasing habitat complexity, which in turn can enhance local herbivore abundance (Clark & Edwards, 1999; Einbinder et al., 2006). It is well documented that concrete structures with many holes can support high concentrations of coral reef fishes (Molles Jr , 1978; Hixon & Beets, 1993; Carr & Hixon, 1997), reflecting patterns on natural reefs (Madin et al., 2019; Santano et al., 2021). Concrete (including Portland cement) has also been used as an effective coral settlement substrate in various regions (Clark & Edwards, 1999; Leonard et al., 2022), including Hawaiʻi (Fitzhardinge & Bailey-Brock, 1989). Artificial structures have been constructed to provide platforms for coral outplanting and to grow fragments for coral nurseries (Forsman et al., 2018; Lange et al., 2024). However, empirical studies are lacking on the mechanisms by which concrete structures, especially those providing shelter for herbivores, may directly or indirectly enhance the recruitment, survival, and growth of juvenile corals.

Abundances of herbivorous fishes around the island of Oʻahu are the lowest in the State of Hawaiʻi (Donovan et al., 2023), calling into question whether herbivores can still provide benefits for corals there. We therefore conducted the Coral Resilience Module Experiment (CReME), on Oʻahu, Hawaiʻi, to assess two key factors affecting the demography of juvenile corals recruiting to concrete modules: shelter availability for herbivores and the relative state of the local reefscape. We compared patterns at a degraded reefscape offshore of densely populated Waikı¯kı¯ with patterns at the fully protected and relatively healthy reefscape at Hanauma Bay. We used standardized roughly cubic-meter concrete settlement modules providing either low- or high-shelter availability for herbivores. These modules allowed us to identify and track naturally settling corals approximately every three months (quarterly) for nearly four years post-deployment, thereby conducting the longest quarterly recruit and juvenile coral demographic monitoring effort on artificial structures of which we are aware.

Our factorial experimental design tested three hypotheses regarding the mechanisms enhancing or inhibiting coral colonization under different environmental conditions. First, the Herbivore Hypothesis proposed that increasing herbivore abundance directly benefits recruit and juvenile corals at local spatial scales, in our study at the scale of experimental modules (Mumby & Steneck, 2008; Hixon, 2015; Williams et al., 2019). This foundational hypothesis predicted that algal overgrowth of corals will vary inversely with herbivore biomass, which will consequently enhance recruitment, survival and growth of juvenile corals. As an extension of this hypothesis, the Shelter Hypothesis asserts that higher shelter availability for herbivores indirectly benefits recruit and juvenile corals independent of the local reef environment (Mumby & Steneck, 2008; Graham & Nash, 2013; Hixon, 2015). This hypothesis predicts that more abundant shelter increases herbivore biomass locally, therefore decreasing algal overgrowth and increasing recruitment, survival, and growth of juvenile corals. Also tested (and not mutually exclusive) was the Reefscape Hypothesis that, independent of shelter availability, relatively healthy reef environments (described below) benefit recruit and juvenile corals (Vermeij & Sandin, 2008; Da-Anoy et al., 2017). This hypothesis predicts that, compared to modules near degraded Waikı¯kı¯ reefs, modules near relatively healthy reefs at Hanauma Bay would experience greater herbivore biomass, lower algal overgrowth, and greater recruitment, survival, and growth of juvenile corals. Some of the results of this field experiment were consistent with all three hypotheses, yet by and large, the hypotheses were rejected. We discuss how a combination of regional overfishing of herbivores, fine-scale environmental variation, and permit-imposed low sample sizes may have caused these patterns, indicating a need for herbivore replenishment to occur hand-and-hand with future coral restoration efforts. Portions of this text were previously published as part of a thesis (Dilley, 2023).

## Methods

### Study sites

**Figure 1:**
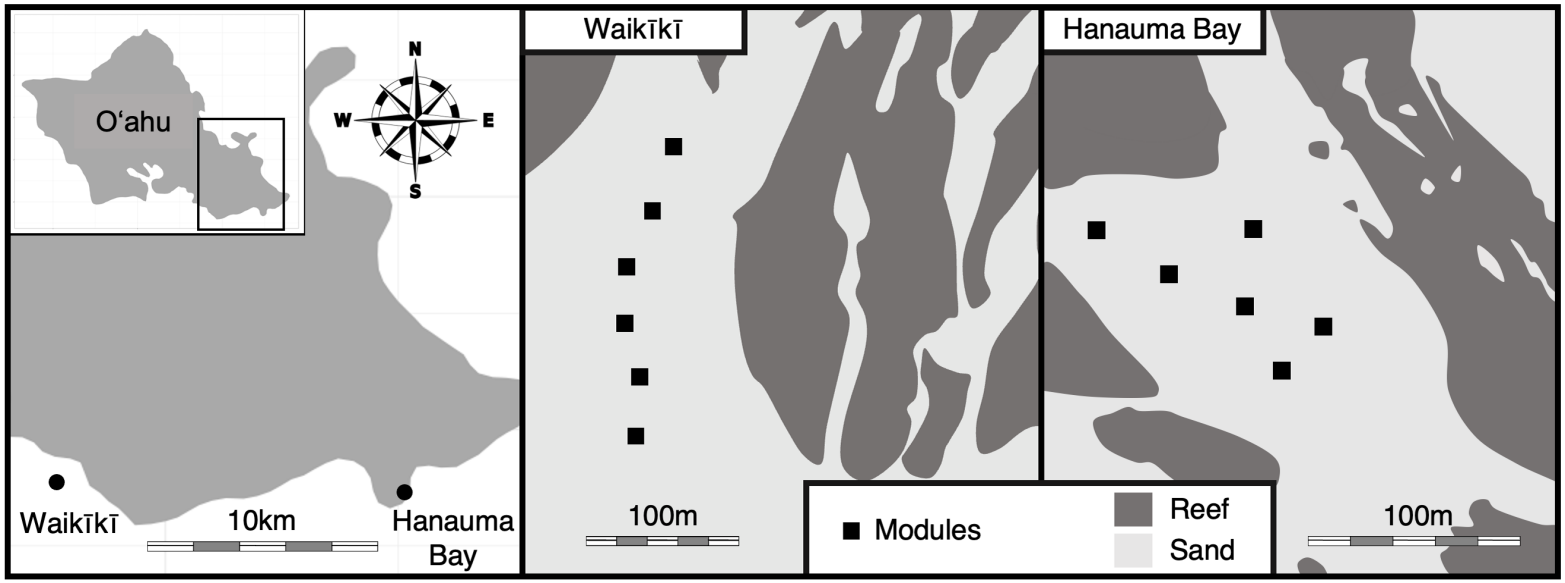
Maps of Waikı¯kı¯ and Hanauma Bay study sites on the south shore of Oʻahu, Hawaiʻi.

Our study was conducted on the south shore of Oʻahu, Hawaiʻi, at two relatively distinct sites: approximately 800 m offshore of Waikı¯kı¯ Beach (21°16′10″N, 157°50′15″W), and at the Hanauma Bay Marine Life Conservation District (21°16′5″N, 157°41′33″W; Fig. 1). Reefs at Waikı¯kı¯ are characterized by spur-and-groove habitat and are adjacent to urban Honolulu, where land-based sources of pollution are concentrated. Coastal waters here have elevated nutrient levels (nitrogen flux = 548.7 kg/day/ha; Wedding et al., 2018) compared to Hanauma Bay from leaching onsite waste disposal systems (Richardson, Dulai & Whittier, 2017). Waikı¯kı¯ reefs have relatively low coral cover of 10% (Franklin, Jokiel & Donahue, 2013), lower fish biomass (Friedlander et al., 2018), especially herbivores (Donovan et al., 2023), due to intensive fishing, and relatively abundant invertebrate coral predators (mostly the cushion seastar, *Culcita novaeguineae* D. Escontrela Dieguez, pers. comm., 2020). Hanauma Bay, a fully protected marine reserve since 1967, consists of continuous reef surrounding large sand patches. This site has substantially lower nutrient levels (nitrogen flux = 39.2 kg/day/ha; Wedding et al., 2018) compared to Waikı¯kı¯, relatively high live coral cover of 30% (Franklin, Jokiel & Donahue, 2013), few invertebrate corallivores (D. Escontrela Dieguez, pers. obs., 2020), and supports greater fish biomass, especially parrotfishes and other herbivores (Friedlander et al., 2018; Donovan et al., 2023).

### Experimental design

The CReME factorial design crossed shelter availability for herbivores (low *vs.* high) and reefscape context (relatively degraded Waikı¯kı¯ *vs.* relatively healthy Hanauma Bay, Fig. S1). All 12 modules were deployed during the summer of 2016 on large sand flats adjacent to reefs, at least 33 m from the nearest natural reef and ranging in depth from 12 to 18 m. Modules were placed on sand due to permitting restrictions to avoid any damage to natural reefs. The coral sand was clean and coarse-grained, and there was neither silt nor natural hard substrata near the modules. Substantial sand scour due to occasional large swells was evident only on the shallowest module at Hanauma Bay, which was consequently excluded from analysis due to very low benthic colonization.

At each site, we interdigitated three low and three high-shelter modules separated from each other by at least 33 m. Each 0.71 m^3^ module was made of 48 standard concrete blocks (each 40 × 19 × 19 cm) that had been soaked in freshwater for 2 weeks and bound together with 316-grade 2-cm stainless-steel banding (Hixon & Beets, 1993; Carr & Hixon, 1997). We tested the effects of shelter availability by constructing modules either with all holes open (high-shelter treatment) or all covered with sheets of concrete fiberboard (low-shelter treatment). The construction-grade fiberboard was inert, like the concrete blocks. Shelter holes of a range of sizes are important for supporting local reef fishes of various body sizes (Hixon & Beets, 1993). The high-shelter modules included 16 tiny (1 × 0.5 cm each), 8 small (9 × 2 cm each), and 24 large holes (15 × 14 cm each) running horizontally through the entire structure (Fig. S2). Low-shelter modules served as a control for any “fish aggregating device” (FAD) effect of fish being attracted to structure regardless of shelter availability.

The exposed edges of the blocks on the hole-free inshore (north) and offshore (south) sides of each module were concave, providing 2-cm deep ledges and overhangs on these settlement surfaces. Thus, each module essentially provided two large, vertical, scalloped concrete settling plates, which did not accumulate sand or other sediment. Overall, the experimental design created comparisons of high and low-shelter treatments at both a degraded reef and a relatively healthy reef in the same habitats at the same depths and the same time.

### Coral surveys

Recruit and juvenile coral demography was quantified by tracking all individual coral colonies that settled on the vertical north and south walls (Fig. S3) of the modules (ca. 1.5 m^2^ combined). Corals were censused quarterly from May 2017 (about one year after module deployment) to March 2020 (COVID outbreak). Divers used Sola Nightsea UV dive lights (Light & Motion) and yellow visors to visualize coral fluorescence, which facilitated finding new settlers as small as a single polyp (Martinez & Abelson, 2013; Leonard et al., 2022). Recruitment was quantified quarterly by counting all new colonies ≤ 5 mm in diameter and dividing by the total available settlement area (*i.e.,* newly observed colonies per m^2^). Survival was calculated as the percentage of colonies that were alive at the end compared to the beginning of each quarter. Colony growth was calculated based on colony maximum diameter (D_m_) measurements to the nearest millimeter *in situ* using a transparent ruler. Maximum orthogonal diameter (D_o_) was also measured *in situ* for a subset of colonies (*Pocillopora*: *n* = 234; *Montipora*: *n* = 51; *Porites*: *n* = 21). For all other colonies, an estimated D_o_ was modelled as a function of D_m_ based on these representative measurements (*Pocillopora*: *r*^2^ = 0.290, *Montipora*: *r*^2^ = 0.833, *Porites*: *r*^2^ = 0.782). D_o_ was modelled to account for differences in growth patterns between the three focal genera. Individual colony planar area was estimated based on an ellipse including the D_m_ and D_o_ values for each colony. Colony growth was calculated as the change in planar area at the end compared to the beginning of each quarter (cm^2^/quarter). Colonies that eventually fragmented, fused, or had shrunk in the previous quarter were excluded from all analyses to differentiate recovery from growth. However, all measurements that preceded these special cases were analyzed.

### Herbivorous fish and urchin surveys

During each survey, we counted and sized to the nearest centimeter all species of herbivorous fishes (TL) and urchins (test diameter) within one meter of the module for approximately 10 min. This included any transient herbivorous fishes that departed the modules as divers approached or visited during the census. All herbivore size estimates were converted to biomass based on published length-weight relationships for fishes (Letourneur, Kulbicki & Labrosse, 1998; Kamikawa et al., 2015; Nadon et al., 2015) and urchins (Lewis, Smith & Eynaud, 2018).

### Benthic algal overgrowth estimates

Algal overgrowth provided an index for coral-algal competition at the colony scale, where negative effects of algae are concentrated, and was highly correlated with overall algal percent cover on module sides (*p* = 0.005, *r*^2^ = 0.167). Starting in the third quarterly coral census (November 2017), overgrowth was assessed for each colony by visually estimating the percentage of each coral colony covered by benthic algae, categorized into quartiles (1: 0–25%, 2: 26–50%, 3: 51–75%, 4: 76–100%). Algal overgrowth was quantified as an index to aid observers in quickly estimating overgrowth of each colony. For analysis, algal overgrowth per module was quantified as the mean overgrowth of all coral colonies on the module (both inshore and offshore sides combined) for each survey period.

### Statistical analyses

To test the Herbivore Hypothesis, we used the *lmer* function in the R package “lme4” (Bates et al., 2015) to determine whether algal overgrowth correlated negatively with herbivore biomass (urchin and herbivorous fish analyzed separately), as well as whether coral recruitment and growth correlated positively with herbivore biomass and negatively with algal overgrowth (Table S1). Coral survival was analyzed using the *glmmTMB* function fitted with a binomial distribution in the R package “glmmTMB” (Brooks et al., 2017) to best fit proportional survivorship data and determine if coral survival correlated positively with herbivore biomass and negatively with algal overgrowth. Model means for all predictors and response variables were calculated on the module scale, matching the scale at which herbivore observations were made (Table S1). For each coral genus, recruitment, survival and growth models were run by binning colonies into four size classes covering recruits and small juvenile corals (1: 1–5 mm, 2: 6–10 mm, 3: 11–15 mm, and 4: 16–20 mm maximum diameter). For all Herbivore Hypothesis models, random effects for module and time were incorporated to account for repeated quarterly measures (Table S1). Survival model fits were inspected using Q-Q plots from the R package “DHARMa” (v0.4.7) whereas all other model fits were inspected using Q-Q plots from the R package “stats” (R Core Team, 2025).

To test the Shelter and Reefscape Hypotheses regarding how herbivore biomass, benthic algal overgrowth, and coral demographic metrics differed between shelter treatments and between study sites, respectively, a series of *glmmTMB*, *clmm*, and *lmer* models were used (Table S1). Urchin and herbivorous fish biomass were analyzed using the *glmmTMB* function fit with a “tweedie” distribution to best fit skewed data with zero inflation. Algal overgrowth was analyzed using the *clmm* function in the R package “ordinal” (Christensen, 2023) to properly account for the ordinal nature of algal overgrowth data (values 1–4). Coral survival was analyzed using the *glmmTMB* function fit with a binomial distribution to best fit proportional survivorship data. Coral recruitment and growth were analyzed using the *lmer* function with coral recruitment values being log(x + 1) transformed to meet assumptions of normality and homoscedasticity. Model means for urchin biomass, herbivorous fish biomass, coral recruitment, and coral survival were calculated at the module scale, whereas model means for algal overgrowth and coral growth were calculated at the coral colony scale. For all Shelter and Reefscape Hypothesis models, random effects for module and time were incorporated to account for repeated quarterly measures. For coral growth, a random effect for coral ID was also used to account for repeated measures at the colony scale (Table S1). Urchin biomass, herbivorous fish biomass, and survival model fits were inspected using Q-Q plots from the R package “DHARMa” whereas all other model fits were inspected using Q-Q plots from the R package “stats”.

To illustrate patterns of survival and growth in greater detail, demographic transition matrices were constructed to determine probabilities of growth, stasis, shrinkage, or death within each size class for each coral genus. Mean transition matrices were calculated according to Doropoulos et al. (2015) and represent mean probabilities of size class transitions per quarter throughout the study. Sample sizes (number of colonies) for each size class are provided at the bottom of each transition matrix (n) in the (Tables S4–S9). Additionally, all sample sizes for linear mixed effects models in this study are provided at the bottom of each model table in the Supplementary Materials (Tables S10–S19).

## Results

The abundances of urchins and herbivorous fishes colonizing the modules were variable through time (Figs. S4A & S4B, respectively) and species observed were representative of surrounding natural reefs (Table S2). Urchins mostly appeared as new recruits, especially in Hanauma Bay early during the study. Almost all resident herbivorous fishes colonized the modules as juvenile and adult immigrants. We occasionally observed schools of large surgeonfishes (*Acanthurus dussumieri* and *A. xanthopterus*) visiting modules at Hanauma Bay, yet never at Waikı¯kı¯. Very few recent settlers (≤ 4 cm) of herbivorous fishes (surgeonfishes and parrotfishes) were observed in our herbivore surveys during the 4-year study, with only 16 individuals seen on the 12 modules and only 39 individuals on 12 comparable natural patch reefs surveyed in parallel with this study (Table S2).

Modules generally had greater bare substrate and less cover of animal benthos at Hanauma Bay than at Waikı¯kı¯ (Table S3). Settling corals were primarily of three common genera: *Pocillopora*, *Montipora*, and *Porites*. Identification to species of recent settlers consisting of only several polyps was not possible *in situ*, yet larger colonies on both modules and natural reefs at both sites were dominated by *Pocillopora meandrina*, *Montipora capitata, M. patula*, and *Porites lobata*.

### Herbivore Hypothesis

This foundational hypothesis tested whether herbivores would benefit recruit and juvenile corals directly by reducing benthic algal overgrowth, thereby enhancing coral recruitment, survival, and growth. Although neither urchin biomass (t = −0.83, *p* = 0.407) nor herbivorous fish biomass (t = −0.34, *p* = 0.731) were significant predictors of mean algal overgrowth, both herbivorous fish biomass and algal overgrowth were significant predictors of coral demographic metrics (Table 1).

**Table 1:** Summary of linear mixed effects model results by coral genus for the herbivore, shelter, and reefscape hypotheses. The Herbivore Hypothesis predicted that herbivore biomass would be positively correlated, and algal overgrowth would be negatively correlated with coral recruitment (Rec), survival (Sur), and growth (Grow). The Shelter Hypothesis predicted that all demographic metrics would be greater on high-shelter (HIGH) modules than on low-shelter (LOW) modules. The Reefscape Hypothesis predicted that all metrics would be greater at relatively healthy Hanauma Bay (HAN) than at relatively degraded Waikı¯kı¯ (WAI). For the latter two hypotheses, model results are presented for both treatments combined (columns labeled “both”). Superscripts denote coral colony size classes (defined in Methods) in which survival and growth correlations were significant (recruitment is by definition to size class 1, so no superscript is necessary).

	Coral Genus								
	*Pocillopora*			*Montipora*			*Porites*		
**Herbivore Hypothesis** predicts correlations positive with herbivores & negative with algae									
Metric:	Rec	Sur	Grow	Rec	Sur	Grow	Rec	Sur	Grow
Urchin herbivores:	0	0	0	0	0	0	0	0	0
Fish herbivores:	0	0	0	0	√^3^	√^4^	0	√^1,2^	0
Algal overgrowth:	√	√^1,4^	0	0	√^4^	0	0	0	0
**Shelter Hypothesis** predicts HIGH >LOW shelter									
Site :	WAI	HAN	both	WAI	HAN	both	WAI	HAN	both
Recruitment:	0	√	0	0	0	0	0	0	0
Survival:	0	0	√^1^	0	0	0	0	√^1^	√^2,3^
Growth:	0	0	0	0	0	0	<^2^	0	<^1^
**Reefscape Hypothesis** predicts HAN >WAI site									
Shelter :	LOW	HIGH	both	LOW	HIGH	both	LOW	HIGH	both
Recruitment:	0	0	0	0	0	√	0	0	0
Survival:	0	0	√^2,3,4^	0	0	0	0	0	0
Growth:	0	0	<^1^	0	0	√^2^	0	0	0

**Notes.**

Symbols √ prediction significantly confirmed 0 no significant difference < results significantly opposite predicted

See Results for details.

We predicted that coral demographic metrics would be increased with greater urchin and herbivorous fish biomass. *Montipora* survival for size class 3 (*p* = 0.018, *r*^2^ = 0.232) and growth for size class 4 (*p* = 0.006, *r*^2^ = 0.121) as well as *Porites* survival for size classes 1 and 2 (1: *p* = 0.018, *r*^2^ = 0.928, 2: *p* = 0.047, *r*^2^ = 0.976) were greater with higher herbivorous fish biomass. Urchin biomass was not correlated with any coral demographic metrics.

We also predicted that coral demographic metrics would be decreased with greater algal overgrowth. *Pocillopora* recruitment (*p* < 0.001, *r*^2^ = 0.063) and survival for size classes 1 and 4 (1: *p* = 0.024, *r*^2^ = 0.127, 4: *p* = 0.046, *r*^2^ = 0.248) as well as *Montipora* survival for size class 4 (*p* = 0.012, *r*^2^ = 0.995) were lower with greater algal overgrowth.

### Shelter Hypothesis

This derivative hypothesis predicted that structural shelter would benefit recruit and juvenile corals indirectly by increasing herbivore biomass locally, thereby decreasing algal overgrowth and enhancing coral demographic metrics. Results mostly confirmed the positive relationship between shelter and herbivores, yet seldom translated to reducing algae and benefitting corals (Table 1).

**Figure 2:**
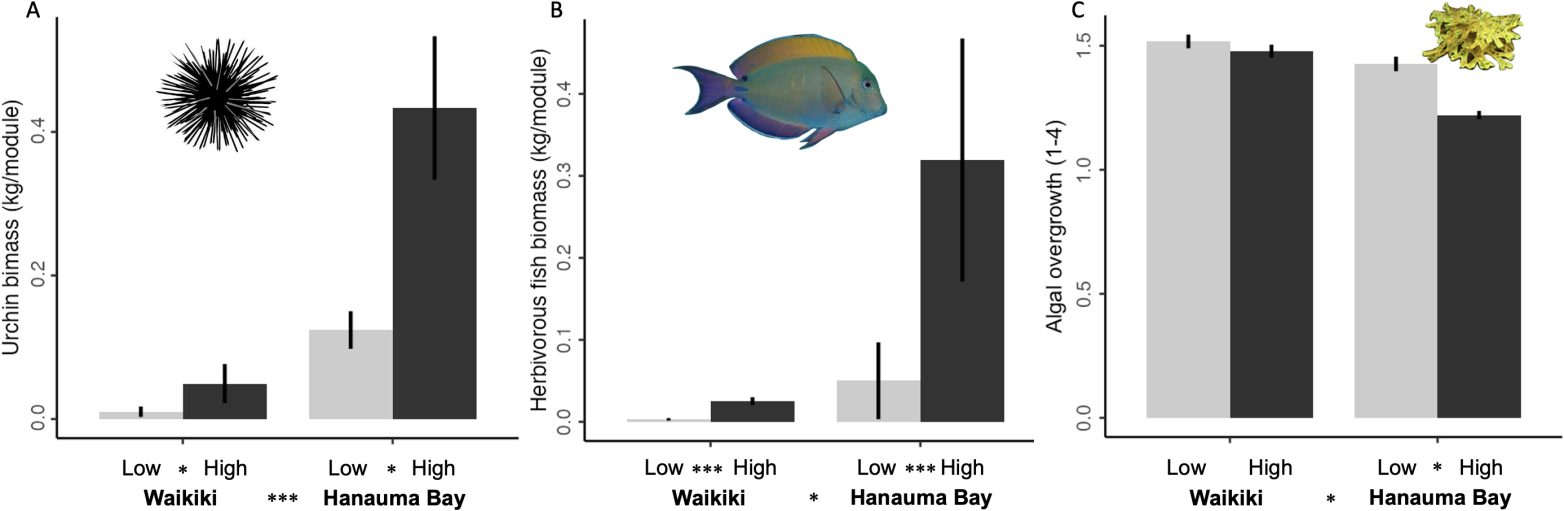
Mean herbivore biomass and benthic algal overgrowth for each site (Waikı¯kı¯ *vs.* Hanauma Bay) and shelter (low *vs.* high) treatment combination. (A) Urchin biomass, (B) herbivorous fish biomass, and (C) benthic algal overgrowth of coral colonies (1: 0–25%, 2: 26–50%, 3: 51–75%, 4: 76–100%) among the four experimental treatments averaged throughout the study (grand means with standard error bars, *n* = 3 modules each, except for one high-shelter module excluded from Hanauma Bay). Asterisks between shelter comparisons (low and high) and site comparisons (Waikı¯kı¯ and Hanauma Bay) indicate significant differences ( ^∗^*p* < 0.05, ^∗∗∗^*p* < 0.001, see text for model results). Algal overgrowth is defined in the text.

Urchin biomass (*p* = 0.002, *r*^2^ = 0.532; Fig. 2A) and herbivorous fish biomass (*p* < 0.001, *r*^2^ = 0.473; Fig. 2B) were greater on high-shelter modules regardless of study site. As expected with greater herbivore biomass, benthic algal overgrowth was lower on average on high-shelter modules (*p* = 0.029, *r*^2^ = 0.045; Fig. 2C).

**Figure 3:**
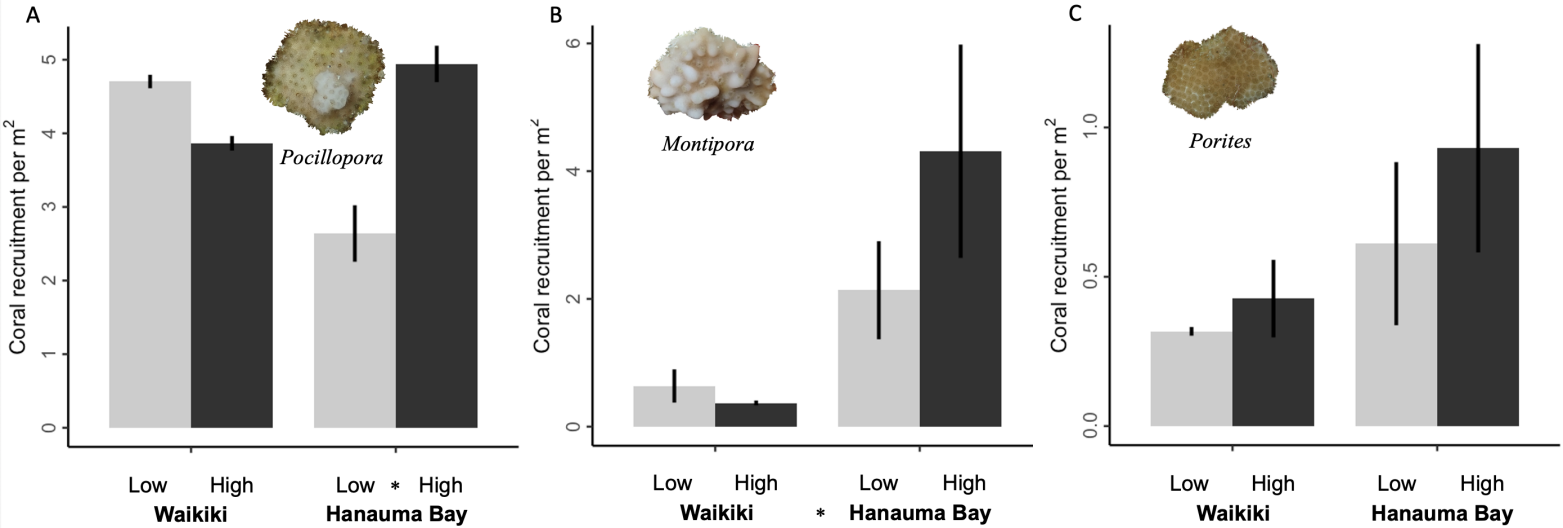
Mean coral recruitment by genus for each site (Waikı¯kı¯ *vs.* Hanauma Bay) and shelter (low *vs.* high) treatment combination. Coral recruitment (number of new colonies per square meter) of (A) *Pocillopora*, (B) *Montipora*, and (C) *Porites* among the four experimental treatments averaged throughout the study (grand means with standard error bars, *n* = 3 modules each, except for one high-shelter module excluded from Hanauma Bay). Note that the graphs are scaled differently. Asterisks between specific shelter comparisons (low and high) and site comparisons (Waikı¯kı¯ and Hanauma Bay) indicate significant differences (^∗^*p* < 0.05, see text for model results).

Given the above patterns, several predictions regarding coral demographic metrics were confirmed. Shelter was a significant predictor of coral recruitment only for *Pocillopora*, with greater values on high-shelter modules only at Hanauma Bay (*p* = 0.01, *r*^2^ = 0.037; Fig. 3A & Fig. S5A). Recruitment was not significantly different between shelter treatments for *Montipora* or *Porites*, despite in most cases being greater on average on high-shelter modules (Figs. 3B & 3C, respectively).

**Figure 4:**
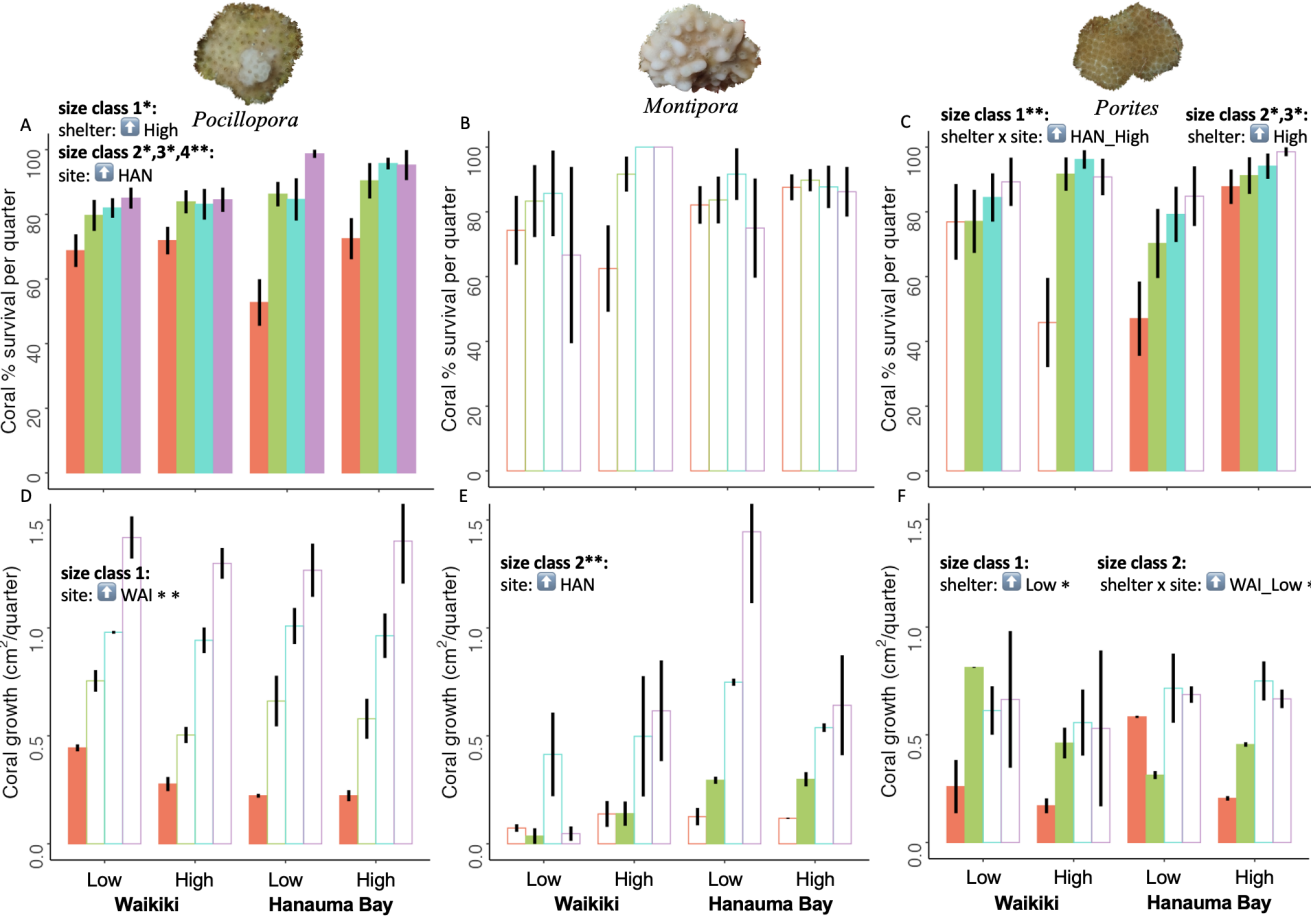
Mean coral survival and growth by genus for each site (Waikı¯kı¯ *vs.* Hanauma Bay) and shelter (low *vs.* high) treatment combination. Quarterly survival of (A) *Pocillopora*, (B) *Montipora*, and (C) *Porites*, as well as quarterly growth of (D) *Pocillopora*, (E) *Montipora*, and (F) *Porites*, among the four experimental treatments averaged throughout the study (grand means with standard error bars, *n* = 3 modules each, except for one high-shelter module excluded from Hanauma Bay). Survival and growth were calculated separately for each size class (1: 1–5 mm: red, 2: 6–10 mm: green, 3: 11–15 mm: blue, 4: 16–20 mm: purple). Bars outlined in bold indicate size classes where significant differences were detected. Size-class specific significant differences between shelter (low *vs.* high) and site (WAI = Waikı¯kı¯ , HAN = Hanauma Bay) treatments, including interactions, are also indicated: upward arrow indicates that treatment was significantly greater (^∗^*p* < 0.05, ^∗∗^*p* < 0.01, see text for statistical test results).

**Figure 5:**
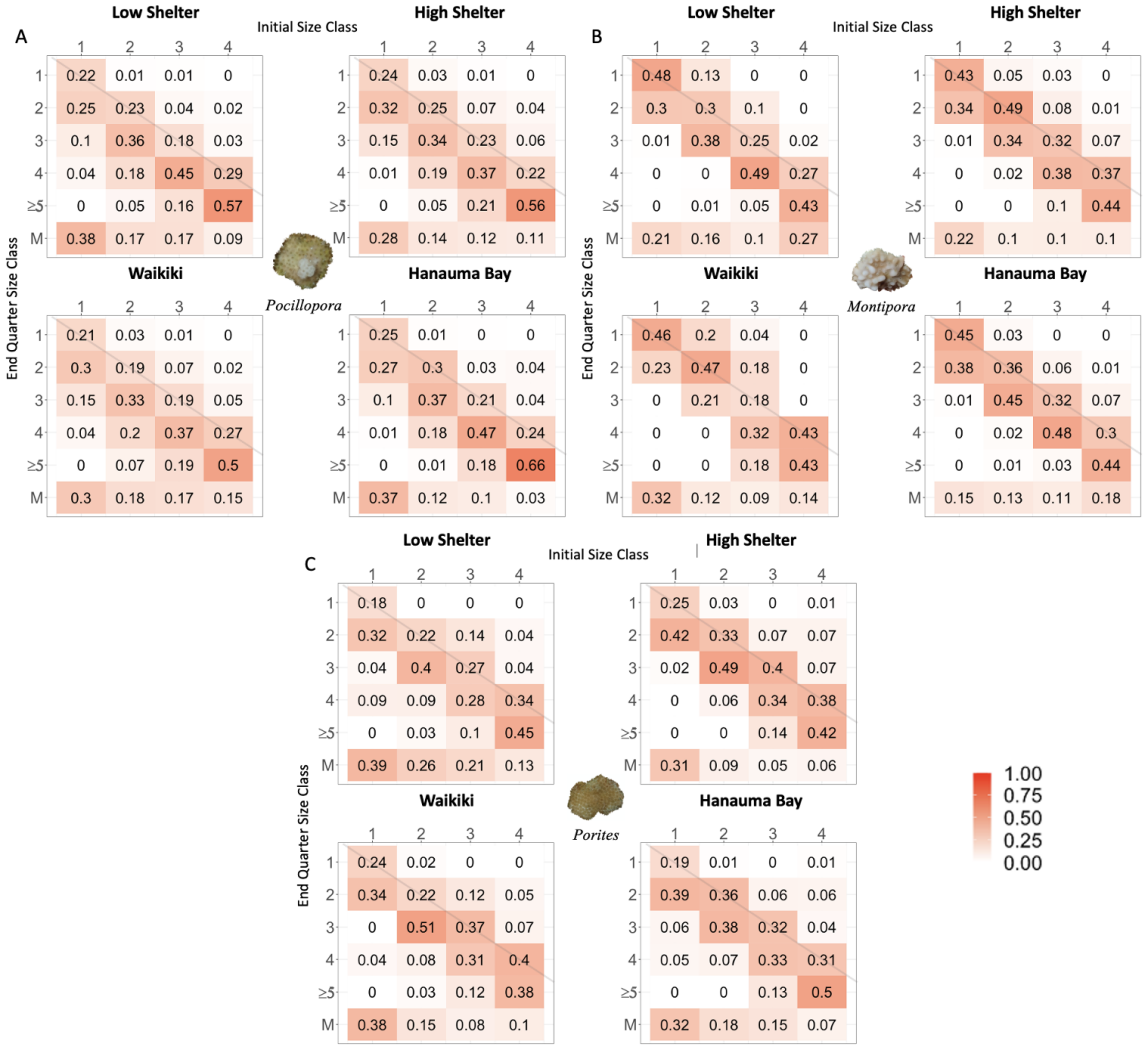
Coral heat maps by genus reflecting demographic transition matrices for each shelter (low *vs.* high) and site (Waikı¯kı¯ *vs.* Hanauma Bay) treatment. Heat maps for (A) *Pocillopora*, (B) *Montipora*, and (C) *Porites* comparing low-shelter *vs.* high-shelter and Waikı¯kı¯ *vs.* Hanauma Bay throughout the experiment. Columns represent the five mm diameter size class at the beginning of a quarter while rows represent the size class at the end of the quarter. Cells contain mean probabilities of either growth (below the diagonal line), stasis (along the diagonal), or shrinkage (above the diagonal) for each possible size-class transition. The second to last row shows cases where corals grew to greater than 20 mm in diameter. The last row (M) shows percent mortality during each transition, such that the sum of values in each column equals 1.0.

As predicted, *Pocillopora* survival was greater on high-shelter modules for size class 1 (*p* = 0.013, *r*^2^ = 0.13; Figs. 4A & 5A) and *Porites* survival was greater on high-shelter modules for size class 1 at Hanauma Bay only (*p* = 0.001, *r*^2^ = 0.282; Fig. 4C, Table S9), where it suffered far greater mortality on low-shelter modules. *Porites* survival was also greater on high-shelter modules for size classes 2 and 3 at both sites (2: *p* = 0.03, *r*^2^ = 0.177, 3: *p* = 0.05, *r*^2^ = 0.137; Figs. 4C & 5C). In contrast, survival of *Montipora* was not significantly different between shelter treatments.

Opposite our predictions, *Porites* grew faster on low-shelter compared to high-shelter modules for size class 1 at both sites (*p* = 0.031, *r*^2^ = 0.127; Figs. 4F & 5C) and for size class 2 at Waikı¯kı¯ only (*p* = 0.026, *r*^2^ = 0.078; Fig. 4F, Table S9). *Pocillopora* and *Montipora* growth was not significantly different between shelter treatments.

### Reefscape Hypothesis

This alternative (yet not mutually exclusive) hypothesis predicted that, independent of shelter treatments, modules near relatively healthy reefs at Hanauma Bay would experience greater herbivore biomass, lower algal overgrowth, and enhanced coral demographic metrics compared to modules near degraded Waikı¯kı¯ reefs. As predicted, urchin biomass (*p* < 0.001, *r*^2^ = 0.532; Fig. 2A) and herbivorous fish biomass (*p* < 0.001, *r*^2^ = 0.473; Fig. 2B) were greater on modules at Hanauma Bay. Also as predicted, benthic algal overgrowth was lower on modules at Hanauma Bay compared to Waikı¯kı¯ (*p* < 0.001, *r*^2^ = 0.045; Fig. 2C).

Despite the above patterns, demographic benefits for corals were seldom detected (Table 1). As expected, *Montipora* recruitment was greater at Hanauma Bay (*p* = 0.01, *r*^2^ = 0.16; Fig. 3B). In contrast, recruitment of *Pocillopora* and *Porites* was not significantly different between the two study sites (Figs. 3A & 3C, respectively).

As predicted, survival of *Pocillopora* was greater at Hanauma Bay for size classes 2, 3, and 4 (2: *p* = 0.035, r^2^ = 0.107, 3: *p* = 0.05, *r*^2^ = 0.179; 4: *p* = 0.005, *r*^2^ = 0.493; Figs. 4A & 5A). However, survival of both *Montipora* and *Porites* was not significantly different between sites (Figs. 4B & 4C, respectively).

Opposite our predictions, growth of *Pocillopora* was greater at Waikı¯kı¯, particularly on low-shelter modules for size class 1 (*p* = 0.005, *r*^2^ = 0.032; Fig. 4D, Table S7). Nonetheless, as predicted, growth of *Montipora* was greater at Hanauma Bay for size class 2 (*p* = 0.004, *r*^2^ = 0.05; Figs. 4E & 5B). Growth of *Porites* was not significantly different between the two study sites (Fig. 4F).

## Discussion

The results of this multiyear field experiment revealed that a complex interplay of environmental factors affected recruit and juvenile corals, varying among genera, shelter treatment, and study site. Although partially corroborated, the predictions of all three *a priori* hypotheses we tested were largely rejected in terms of demographic metrics of newly settled corals. There is evidence that this outcome was due to a combination of severe overfishing of herbivores around Oʻahu (Donovan et al., 2023) as well as fine-scale environmental variation, exacerbated by low sample sizes (dictated by the permitting process in Hawaiʻi). Despite the resulting reduced power of our statistical analyses, some significant patterns emerged nonetheless.

### Herbivore Hypothesis

The foundational hypothesis we tested, widely confirmed outside Hawaiʻi (review by Hixon, 2015), was that herbivores directly benefit recruit and juvenile corals by cropping or removing nearby algae. Overall, we found that herbivore biomass was not a significant predictor of algal overgrowth, even though urchin and herbivorous fish biomass were greater and algal overgrowth was lower on Hanauma Bay modules. During this study, herbivorous fish and urchins were occasionally observed moving between modules and the surrounding natural reef. It is therefore likely that transient herbivore biomass was underestimated during this study and may have affected our ability to detect a negative correlation between herbivore biomass and algal overgrowth.

Consistent with the Herbivore Hypothesis, a few genus-specific coral demographic metrics were found to be positively and negatively correlated with herbivorous fish biomass and algal overgrowth respectively. *Montipora* survival for size class 3 and growth for size class 4 as well as *Porites* survival for size classes 1 and 2 were positively correlated with herbivorous fish biomass. Additionally, *Pocillopora* recruitment and survival for size classes 1 and 4 as well as *Montipora* survival for size class 4 were negatively correlated with benthic algal overgrowth. These patterns suggest that, despite the lack of a negative correlation between herbivore biomass (urchins and herbivorous fishes) and algal overgrowth, greater herbivore biomass and lower algal overgrowth benefitted recruit and juvenile corals regardless of site or shelter availability for herbivores. Future *in situ* studies on the effects of herbivory on recruit and juvenile coral demographic metrics would benefit from quantifying both resident and transient herbivore biomass. Remote video surveys can provide transient herbivore frequency of visitation and biomass data, which combined with biomass from resident herbivores, can provide better estimates of overall herbivory. Additionally, future work on recruit and juvenile coral mortality should focus on coral-algal interactions and how overgrowth by different algal taxa affect varying sized corals of specific species differently. These insights would provide valuable context to explain coral survival and growth for different coral species with varying levels of herbivory and algal overgrowth.

### Shelter Hypothesis

This extension of the Herbivore Hypothesis predicted that spatial refugia for herbivores indirectly benefit recruit and juvenile corals (regardless of reefscape). Although herbivore biomass was greater and algal overgrowth was lower on high-shelter modules, particularly at Hanauma Bay, this hypothesis was also mostly rejected. It appears that this hypothesis failed because of the generally low abundance of herbivores in our study system. Herbivorous fishes, such as parrotfishes and surgeonfishes, are severely overfished on Oʻahu and parts of other main Hawaiian Islands (Nadon, 2017; Friedlander et al., 2018; Donovan et al., 2023). Reef fish populations in Hawaiʻi may be additionally suppressed due to recruitment limitation (Walsh, 1987), which was clearly evident in the extremely low number of recently recruited herbivores we observed over four years. Regarding urchins, typical of coral-reef species globally, including the genera encountered during our study (Birkeland, 1989), recruitment was patchy in time and space, occurring mostly in the beginning of this study on Hanauma Bay modules. With few herbivores in general, the direct and indirect effects of structural shelter were obscured relative to general patterns found elsewhere in the world (Mumby & Steneck, 2008; Graham & Nash, 2013; Hixon, 2015).

While most demographic predictions of the Shelter Hypothesis were rejected, a few were confirmed. First, *Pocillopora* recruitment was greater on high-shelter modules, yet this pattern occurred only at Hanauma Bay. Counterintuitively, Waikı¯kı¯ modules (both low- and high-shelter) had considerable *Pocillopora* recruitment despite module surfaces there having dense algal turfs and greater cover of space competitors like sponges and tunicates. This result may be due to a potentially greater abundance of *Pocillopora* larvae at Waikı¯kı¯ (Tsounis & Edmunds, 2016), where this genus dominates as a quick colonizer on the surrounding natural reefs, compared to Hanauma Bay where *Porites* is dominant (Grigg, 1998). Perhaps because of high larval availability, *Pocillopora* colony density was comparable across Waikı¯kı¯ modules at the end of this study regardless of structural shelter treatments. We therefore now hypothesize that *Pocillopora* benefitted from the exceptionally clean surfaces of high-shelter modules at Hanauma Bay, despite not being a dominant coral genus on surrounding reefs, yet at the same time, benefitted from relatively high larval supply at Waikı¯kı¯, perhaps compensating for poorer settlement habitat at that site.

Second, high-shelter modules at Hanauma Bay exhibited greater *Porites* survivorship, particularly for the smallest size class. Greater *Porites* survival also occurred on high-shelter modules for size classes 2 and 3 regardless of site, despite greater algal overgrowth on Waikı¯kı¯ modules. Sediment retention by thick algal turfs (Nugues & Roberts, 2003) and the presence of algal metabolites and allelochemicals that can create hypoxic conditions (Brown et al., 2020) may be important factors in determining early survivorship of juvenile *Porites*. Water motion that perturbs thick turfs can reduce these effects (Jorissen et al., 2016). Indeed, *Porites* in contact with algal turf has been found to incur greater tissue damage in habitats with low water flow and high sedimentation (Gowan, Tootell & Carpenter, 2014). Therefore, we hypothesize that the many holes in high-shelter modules created conditions of more variable water flow that reduced sediment loads and the negative effects of thick turfs, thereby enhancing the survival of *Porites*.

Reduced survival on low-shelter modules may also be attributed to greater growth in response to intense competition with algal turfs. Contrary to our predictions, *Porites* growth was greater for the smallest colonies on low-shelter modules, and especially at Waikı¯kı¯ for size class 2. We hypothesize that faster growth in response to unfavorable microhabitats within thick algal turfs may impose a metabolic cost that reduces survival (Denis et al., 2013), perhaps involving decreased resistance to pathogens and harmful metabolites associated with the algae (Kuffner et al., 2006; Smith et al., 2006; Evensen et al., 2019).

### Reefscape Hypothesis

As an alternative to the other two herbivore-based hypotheses (yet not mutually exclusive), the Reefscape Hypothesis predicted that a relatively healthy surrounding reef with abundant herbivores and lower algal overgrowth would benefit the demographic metrics of recruit and juvenile corals (regardless of shelter). Like the other hypotheses we tested, most demographic predictions were rejected, yet more were confirmed for the Reefscape Hypothesis than for the Herbivore and Shelter Hypotheses. In particular, recruitment and growth of *Montipora* and survival of *Pocillopora* were greater on experimental modules at Hanauma Bay, where herbivore biomass was greater and algal overgrowth was lower than on modules at Waikı¯kı¯.

Regarding recruitment, *Montipora* showed significantly higher values at Hanauma Bay. There are several possible explanations. First, the relatively high abundance of *Montipora* on the surrounding reef at this site likely enhanced larval supply for settlement on the experimental modules compared to Waikı¯kı¯. Second, lower settlement at relatively algal-laden Waikı¯kı¯ may have been due to microbe-mediated mortality of planulae. Vermeij et al. (2009) demonstrated experimentally in Maui that harmful microbes associated with fleshy algae increased mortality of *Montipora* planulae, which may have contributed to a settlement bottleneck at Waikı¯kı¯. Third, sponges and ascidians were more abundant on modules at Waikı¯kı¯ (Table S3) and are known to reduce coral recruitment (Brandt et al., 2019), which could possibly have affected *Montipora* in particular by some unknown mechanism. Fourth, *Montipora* in Palau was documented to recruit to habitats with abundant crustose coralline algae (CCA; Gouezo et al., 2020), which produce chemical cues that enhance larval settlement (Ritson-Williams et al., 2009). During our study, CCA were rarely seen at Waikı¯kı¯ compared to high-shelter modules at Hanauma Bay where *Montipora* recruitment was much greater (Table S3). Considering that the cleanest settlement surfaces with higher CCA cover were on high-shelter modules at Hanauma Bay, and that the surrounding reef has greater abundance of *Montipora*, it follows that recruitment was far greater there. It is also possible that *Montipora* mortality was greater at Waikı¯kı¯, but the low abundance of recruits there precluded detection of a significant pattern.

Mortality of *Pocillopora* was positively correlated with algal overgrowth for size classes 1 and 4 as well as greater at Waikı¯kı¯ for size classes 2, 3, and 4. Based on these findings, algal overgrowth does appear to reduce *Pocillopora* survival, consistent with prior studies where algae reduces juvenile coral survival (Box & Mumby, 2007; Birrell et al., 2008; Penin et al., 2011). Additionally, *Pocillopora* may have been at a disadvantage relative to the other coral genera because corallivorous cushion seastars (*Culcita novaeguineae*) were more abundant on modules at Waikı¯kı¯ (13 individuals observed) than at Hanauma Bay, where they were never seen during this study. This corallivore prefers pocilloporid corals over other genera in Hawaiʻi (Glynn & Krupp, 1986) and therefore could have differentially reduced the survival of *Pocillopora* juveniles at Waikı¯kı¯. Corallivory was difficult to detect during our rare observations, as telltale evidence of such predation is the white color of coral skeletons, which at the small sizes of new recruits are quickly overgrown with algae. On many occasions at Waikı¯kı¯, *Pocillopora* colonies were overgrown by colonial sponges and ascidians, which had higher coverage there. We therefore hypothesize that greater predation combined with greater algal overgrowth and competition with more abundant sponges and ascidians at Waikı¯kı¯ contributed to higher pocilloporid mortality there.

Regarding growth, *Pocillopora* grew faster at Waikı¯kı¯ for size class 1. Similar to *Porites*, we hypothesize that faster growth of *Pocillopora* at Waikı¯kı¯ was a response to increased algal overgrowth and greater competition with sponges and ascidians at Waikı¯kı¯. Consequently, it is possible that rapid growth in the smallest pocilloporid corals may impose metabolic costs that reduce survival (Denis et al., 2013) and increase susceptibility to pathogens and harmful algal metabolites (Kuffner et al., 2006; Smith et al., 2006; Evensen et al., 2019) similar to our hypothesis that faster growth of *Porites* led to greater mortality on low-shelter modules. This conclusion is supported by the subsequent size classes (2–4) of *Pocillopora* having lower survivorship at Waikı¯kı¯ and with greater algal overgrowth regardless of site.

Conversely, *Montipora* growth for size class 2 was significantly greater at Hanauma Bay compared to Waikı¯kı¯. This result was likely associated with a positive correlation between herbivorous fish biomass and *Montipora* growth, combined with greater herbivorous fish abundance and lower algal overgrowth at Hanauma Bay. Reduced competition with algae and other benthos at Hanauma Bay likely benefitted recruit *Montipora* resulting in faster colony growth (Barrows et al., 2023). Waikı¯kı¯ had very few *Montipora* colonies throughout this study, leading to highly unbalanced sample sizes between the sites. The low abundance of *Montipora* at Waikı¯kı¯ could therefore have obscured our ability to detect significant differences between sites in the growth rates of other size classes.

### Ramifications for coral restoration

The Coral Resilience Module Experiment (CReME) was designed to test whether, given the overfished status of herbivores around the island of Oʻahu, concrete structures can be an effective tool for coral restoration by providing both coral settlement surfaces and shelter for herbivores that benefit corals. Predictions of the Herbivore, Shelter, and Reefscape Hypotheses regarding recruitment, survival, and growth of juvenile corals were largely rejected for three common coral genera, likely due to low densities of herbivorous fishes around Oʻahu, patchy urchin recruitment, and low replication of experimental modules imposed by state and federal permits. The few confirmed predictions preclude broad generalizations. Nonetheless, our factorial experimental design did successfully detect multiple cases of both genus-specific and context-dependent patterns in demographic metrics of recruit and juvenile corals.

We believe that the most important lesson from this study for coral restoration is the importance of management to replenish herbivore populations. Given relatively low recruitment of fishes during our study and elsewhere in Hawaiʻi (Walsh, 1987), and especially that herbivorous fishes are often severely overfished in Hawaiʻi (Nadon, 2017; Friedlander et al., 2018; Donovan et al., 2023), it is imperative that reef managers replenish herbivore populations to enhance coral restoration efforts (Chung et al., 2019a; Gove et al., 2023). The Hawaiʻi Division of Aquatic Resources has successfully cultured collector urchins (*Tripneustes gratilla*) to control invasive algae in Kāneʻohe Bay on Oʻahu (Neilson et al., 2018). Additionally, an herbivore replenishment reserve on Maui has demonstrated that greater herbivore populations can facilitate coral recovery where macroalgae once dominated (Williams et al., 2016). Herbivore management and community-based subsistence fisheries areas (CBSFAs), co-managed by local communities and the State of Hawaiʻi can provide a means to replenish depleted herbivore populations by empowering local communities to develop place-based management strategies with community engagement (Chung et al., 2019a). However, more spatial management is needed to address the scope and scale of herbivorous fish population declines in Hawaiʻi (Chung et al., 2019b).

The clearest result of this experiment was that proximity of relatively healthy coral reefs with high herbivore abundance and low algal cover can enhance coral recruitment, survival, and growth of juvenile corals on coral-restoration modules. This study also clearly confirmed that concrete modules provide adequate settlement surfaces for studying early post-settlement coral demography. Therefore, to effectively restore coral reefs using artificial structures, reef managers should consider placing them in degraded areas with relatively healthy coral and herbivore populations nearby. Greater herbivory from resident and transient herbivores combined with more abundant coral planulae from nearby healthy reefs can enhance both coral outplant survival and natural coral recruitment on artificial structures and consequently facilitate coral population recovery on nearby degraded reefs. We followed hundreds of naturally settled corals on these modules quarterly for nearly four years, uniquely demonstrating the feasibility of long-term and frequent monitoring of recruit and juvenile coral demographic metrics *in situ*. As juvenile corals grow, they become less susceptible to local sources of mortality (Doropoulos et al., 2015), so future research focusing on the demography of recruit and juvenile corals is key to effectively restoring and managing coral populations (Edmunds & Riegl, 2020; Edmunds, Maritorena & Burgess, 2024). Further understanding the mechanisms underlying patterns of recruitment, survival, and growth of juvenile corals will be crucial for reef restoration efforts and enhancing natural coral recovery in a rapidly warming ocean, where worsening bleaching events will certainly challenge the ability of corals to resist and recover from climate disruption (Van Hooidonk et al., 2016).

## Supplemental Information

10.7717/peerj.20891/supp-1 Supplemental Information 1 CReME project experimental design The factorial experimental design of the CReME project, with high and low shelter modules deployed at both Waikı¯kı¯ (relatively degraded reefscape) and Hanauma Bay (relatively healthy reefscape).

10.7717/peerj.20891/supp-2 Supplemental Information 2 Experimental modules onshore before banding and deployment

10.7717/peerj.20891/supp-3 Supplemental Information 3 Representative photos of module sides for both site and shelter treatments Photos near the end of the experiment of concrete modules at the relatively degraded reef of Waikı¯kı¯ (9/26/2019) and the relatively healthy reef of Hanauma Bay (9/24/2019). Turf, macroalgae, and encrusting sessile benthos (*e.g.*, sponges, ascidians, and bryozoans), all of which displace corals, were much more prevalent at Waikı¯kı¯, whereas larger coral colonies were more prevalent at Hanauma Bay.

10.7717/peerj.20891/supp-4 Supplemental Information 4 Herbivore biomass and benthic algal growth time series for each site (Waikı¯kı¯*vs.* Hanauma Bay) and shelter (low *vs.* high) treatment combination Time series plots (mean ±standard error) for (A) urchin biomass, (B) herbivorous fish biomass, and (C) algal overgrowth (*n* = 3 modules, except for one high-shelter module excluded from Hanauma Bay).

10.7717/peerj.20891/supp-5 Supplemental Information 5 Recruitment time series for each site (Waikı¯kı¯ *vs.* Hanauma Bay) and shelter (low *vs.* high) treatment combination Time series plots for recruitment (mean ±standard error, *n* = 3 modules, except for one high-shelter module excluded from Hanauma Bay) for recruitment of (A) *Pocillopora*, (B) *Montipora*, (C) *Porites*, and (D) *Porites* re-scaled for easier interpretation.

10.7717/peerj.20891/supp-6 Supplemental Information 6 Coral colony density shelter and site treatment comparisons on experimental modules Coral colony abundance per meter squared of substrate at the last coral census during the study period (mean ±standard error, *n* = 3 modules, except for one high-shelter module excluded from Hanauma Bay).

10.7717/peerj.20891/supp-7 Supplemental Information 7 R functions and equations used for all study analyses Summary table of R model functions used and equations for each hypothesis. Scale indicates whether response variables are on the module (*e.g.*, recruitment as recruits per m^2^ of substrate of the entire module) or coral colony scale. Random effects for module replicate (1—m), quarter nested in year (1—t), and coral colony identity (1—ID) to account for repeated measures.

10.7717/peerj.20891/supp-8 Supplemental Information 8 Herbivore species observed during this study Herbivorous fishes and urchins observed on experimental modules (6 per site), comparable natural patch reefs (6 per site), and continuous reef transects (*n* = 5) during herbivore surveys at both Waikı¯kı¯ and Hanauma Bay. “X” denotes that at least one individual of that species was observed over 4 yr. “X*” denotes that at least one new recruit fish (four cm TL) of that species was observed.

10.7717/peerj.20891/supp-9 Supplemental Information 9 Percent cover of algae, sessile benthic invertebrates and bare substrate on experimental modules Mean ±standard error percent cover for each benthic taxon and bare space on surveyed module sides (north and south walls combined) for Waikı¯kı¯-Low Shelter, Waikı¯kı¯ -High Shelter, Hanauma Bay-Low Shelter, and Hanauma Bay-High Shelter modules. Based on photographs taken at the beginning and end of the summer from 2017 to 2019 (TimeSteps: 1, 2, 5, 6, 9, 10).

10.7717/peerj.20891/supp-10 Supplemental Information 10 * Pocillopora* transition matrices comparing low shelter *vs.* high shelter and Waikı¯kı¯ *vs.* Hanauma Bay modules throughout the experiment Columns represent the five mm diameter size class at the beginning of a quarter while rows represent the size class at the end of the quarter. Cells contain mean probabilities of either growth (below the diagonal line), stasis (along the diagonal), or shrinkage (above the diagonal) for each possible size class transition with associated standard error. The third to last row shows cases where corals grew to greater than 20 mm in diameter. The second to last row (M) shows percent mortality during each transition, such that the sum of values in each column equals 1.0. The last row (n) represents the total number of observations for each given size class.

10.7717/peerj.20891/supp-11 Supplemental Information 11 * Montipora* transition matrices comparing low shelter *vs.* high shelter and Waikı¯kı¯ *vs.* Hanauma Bay modules throughout the experiment Columns represent the five mm diameter size class at the beginning of a quarter while rows represent the size class at the end of the quarter. Cells contain mean probabilities of either growth (below the diagonal line), stasis (along the diagonal), or shrinkage (above the diagonal) for each possible size class transition with associated standard error. The third to last row shows cases where corals grew to greater than 20 mm in diameter. The second to last row (M) shows percent mortality during each transition, such that the sum of values in each column equals 1.0. The last row (n) represents the total number of observations for each given size class.

10.7717/peerj.20891/supp-12 Supplemental Information 12 * Porites* transition matrices comparing low shelter *vs.* high shelter and Waikı¯kı¯ *vs.* Hanauma Bay modules throughout the experiment Columns represent the five mm diameter size class at the beginning of a quarter while rows represent the size class at the end of the quarter. Cells contain mean probabilities of either growth (below the diagonal line), stasis (along the diagonal), or shrinkage (above the diagonal) for each possible size class transition with associated standard error. The third to last row shows cases where corals grew to greater than 20 mm in diameter. The second to last row (M) shows percent mortality during each transition, such that the sum of values in each column equals 1.0. The last row (n) represents the total number of observations for each given size class.

10.7717/peerj.20891/supp-13 Supplemental Information 13 * Pocillopora* transition matrices for Waikı¯kı¯-Low Shelter, Hanauma Bay-Low Shelter, Waikı¯kı¯-High Shelter, and Hanauma Bay-High Shelter modules Columns represent the five mm diameter size class at the beginning of a quarter while rows represent the size class at the end of the quarter. Cells contain mean probabilities of either growth (below the diagonal line), stasis (along the diagonal), or shrinkage (above the diagonal) for each possible size class transition with associated standard error. The third to last row shows cases where corals grew to greater than 20 mm in diameter. The second to last row (M) shows percent mortality during each transition, such that the sum of values in each column equals 1.0. The last row (n) represents the total number of observations for each given size class.

10.7717/peerj.20891/supp-14 Supplemental Information 14 * Montipora* transition matrices for Waikı¯kı¯-Low Shelter, Hanauma Bay-Low Shelter, Waikı¯kı¯-High Shelter, and Hanauma Bay-High Shelter modules Columns represent the five mm diameter size class at the beginning of a quarter while rows represent the size class at the end of the quarter. Cells contain mean probabilities of either growth (below the diagonal line), stasis (along the diagonal), or shrinkage (above the diagonal) for each possible size class transition with associated standard error. The third to last row shows cases where corals grew to greater than 20 mm in diameter. The second to last row (M) shows percent mortality during each transition, such that the sum of values in each column equals 1.0. The last row (n) represents the total number of observations for each given size class.

10.7717/peerj.20891/supp-15 Supplemental Information 15 * Porites* transition matrices for Waikı¯kı¯-Low Shelter, Hanauma Bay-Low Shelter, Waikı¯kı¯-High Shelter, and Hanauma Bay-High Shelter modules Columns represent the five mm diameter size class at the beginning of a quarter while rows represent the size class at the end of the quarter. Cells contain mean probabilities of either growth (below the diagonal line), stasis (along the diagonal), or shrinkage (above the diagonal) for each possible size class transition with associated standard error. The third to last row shows cases where corals grew to greater than 20 mm in diameter. The second to last row (M) shows percent mortality during each transition, such that the sum of values in each column equals 1.0. The last row (n) represents the total number of observations for each given size class.

10.7717/peerj.20891/supp-16 Supplemental Information 16 Herbivore hypothesis analysis for algal overgrowth *vs.* herbivore biomass Algal overgrowth was analyzed using the lmer function. *σ*^2^ and *t*_00_ represent the residual variance and random effect variance explained respectively.

10.7717/peerj.20891/supp-17 Supplemental Information 17 Herbivore hypothesis analyses for recruitment of *Pocillopora*, *Montipora*, and *Porites* Recruitment was analyzed using the lmer function. Recruitment response variable was log(x + 1) transformed for all three genera. *σ*^2^ and *t*_00_ represent the residual variance and random effect variance explained respectively.

10.7717/peerj.20891/supp-18 Supplemental Information 18 Herbivore hypothesis analyses for survival and growth of *Pocillopora* (PC) Survival was analyzed using the *glmmTMB* function with a binomial distribution whereas growth was analyzed using the lmer function. *σ*^2^ and *t*_00_ represent the residual variance and random effect variance explained respectively.

10.7717/peerj.20891/supp-19 Supplemental Information 19 Herbivore hypothesis analyses for survival and growth of *Montipora* (MO) Survival was analyzed using the *glmmTMB* function with a binomial distribution whereas growth was analyzed using the lmer function. *σ*^2^ and *t*_00_ represent the residual variance and random effect variance explained respectively.

10.7717/peerj.20891/supp-20 Supplemental Information 20 Herbivore hypothesis analyses for survival and growth of *Porites* (PR) Survival was analyzed using the *glmmTMB* function with a binomial distribution whereas growth was analyzed using the lmer function. *σ*^2^ and *t*_00_ represent the residual variance and random effect variance explained respectively.

10.7717/peerj.20891/supp-21 Supplemental Information 21 Shelter and reefscape hypotheses analyses for urchin biomass, herbivorous fish biomass, and algal overgrowth Urchin biomass and herbivorous fish biomass were analyzed using the glmmTMB function with a log link tweedie distribution and algal overgrowth was analyzed using the clmm function for ordinal data. *σ*^2^ and *t*_00_ represent the residual variance and random effect variance explained respectively.

10.7717/peerj.20891/supp-22 Supplemental Information 22 Shelter and reefscape hypotheses analyses for recruitment of *Pocillopora* (PC), *Montipora* (MO), and *Porites* (PR) Recruitment was analyzed using the lmer function. Recruitment response variable was log(x + 1) transformed for all three genera. *σ*^2^ and *t*_00_ represent the residual variance and random effect variance explained respectively.

10.7717/peerj.20891/supp-23 Supplemental Information 23 Shelter and reefscape hypotheses analyses for survival and growth of *Pocillopora* (PC) Survival was analyzed using the *glmmTMB* function with a binomial distribution whereas growth was analyzed using the lmer function. *σ*^2^ and *t*_00_ represent the residual variance and random effect variance explained respectively.

10.7717/peerj.20891/supp-24 Supplemental Information 24 Shelter and reefscape hypotheses analyses for survival and growth of *Montipora* (MO) Survival was analyzed using the *glmmTMB* function with a binomial distribution whereas growth was analyzed using the lmer function. *σ*^2^ and *t*_00_ represent the residual variance and random effect variance explained respectively.

10.7717/peerj.20891/supp-25 Supplemental Information 25 Shelter and reefscape hypotheses analyses for survival and growth of *Porites* (PR) Survival was analyzed using the *glmmTMB* function with a binomial distribution whereas growth was analyzed using the lmer function. *σ*^2^ and *t*_00_ represent the residual variance and random effect variance explained respectively.

## Additional Information and Declarations

### Data Availability

The following information was supplied regarding data availability: The raw data are available at Zenodo: Dilley, E. (2026). Dilley et al. 2026 Data Repository [Data set]. Zenodo. https://doi.org/10.5281/zenodo.18628839.
