# Supplementary figures and images for: Coral restoration: roles of shelter for herbivores and reef state in early recruitment success
Source: PeerJ. 2026 Apr 7;14:e20891. doi: 10.7717/peerj.20891 (PMC13068014; doi:10.7717/peerj.20891)

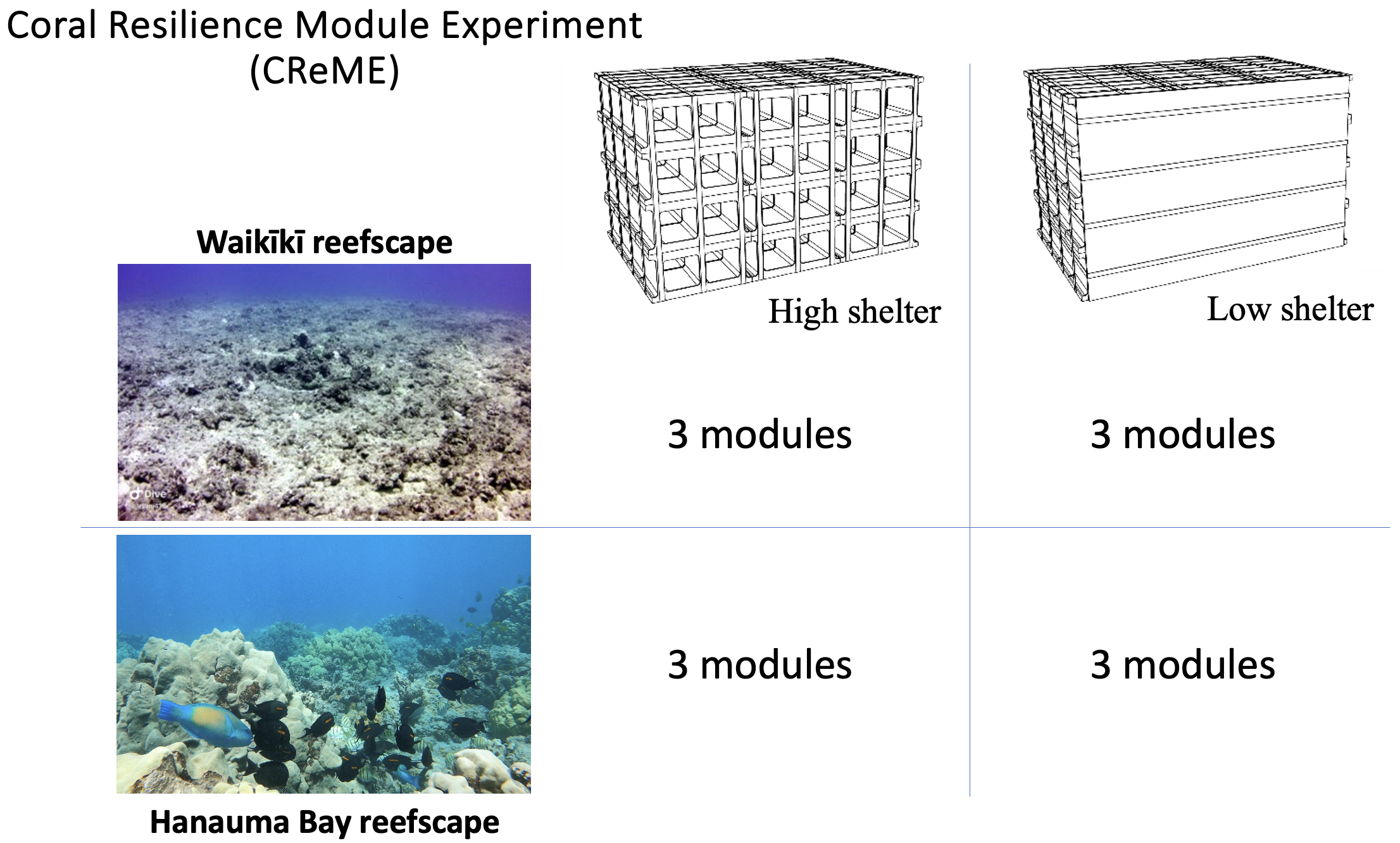

Supplement: Supplemental Information 1 — The factorial experimental design of the CReME project, with high and low shelter modules deployed at both Waikı¯kı¯ (relatively degraded reefscape) and Hanauma Bay (relatively healthy reefscape). [file peerj-14-20891-s001.png]

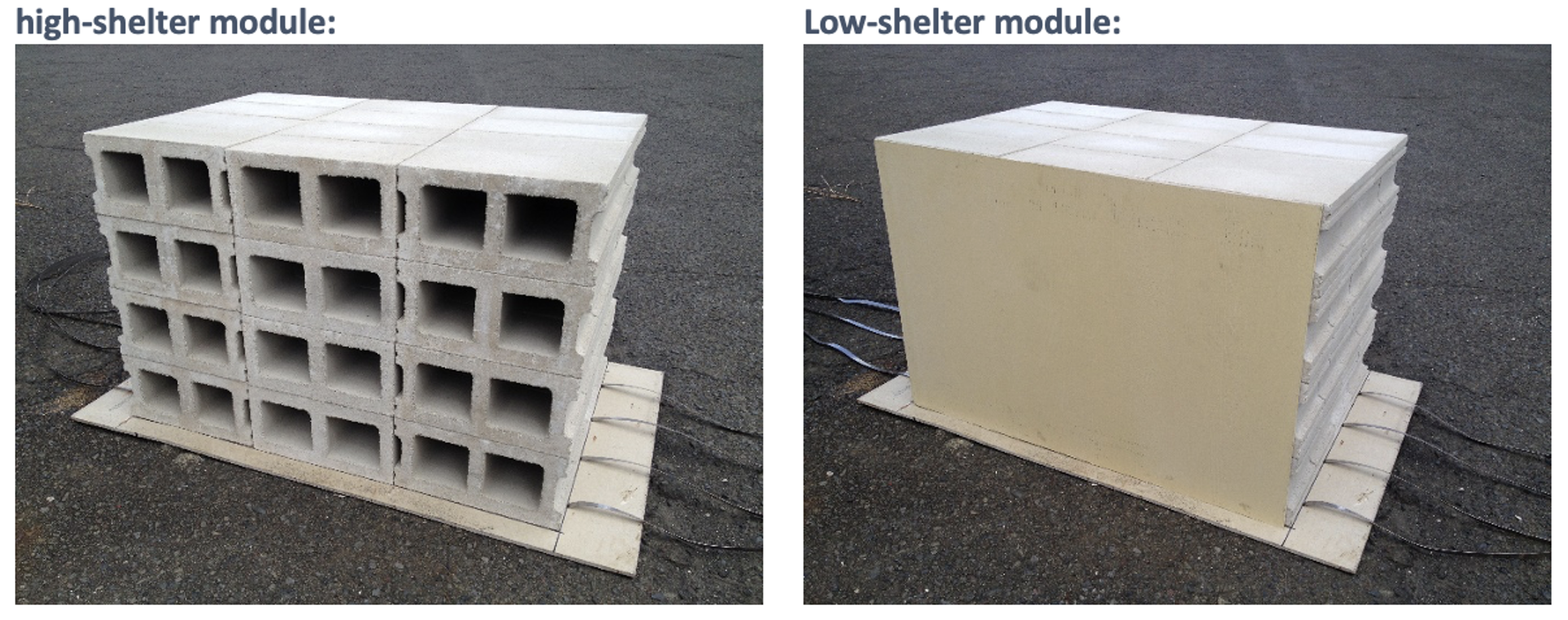

Supplement: Supplemental Information 2 [file peerj-14-20891-s002.png]

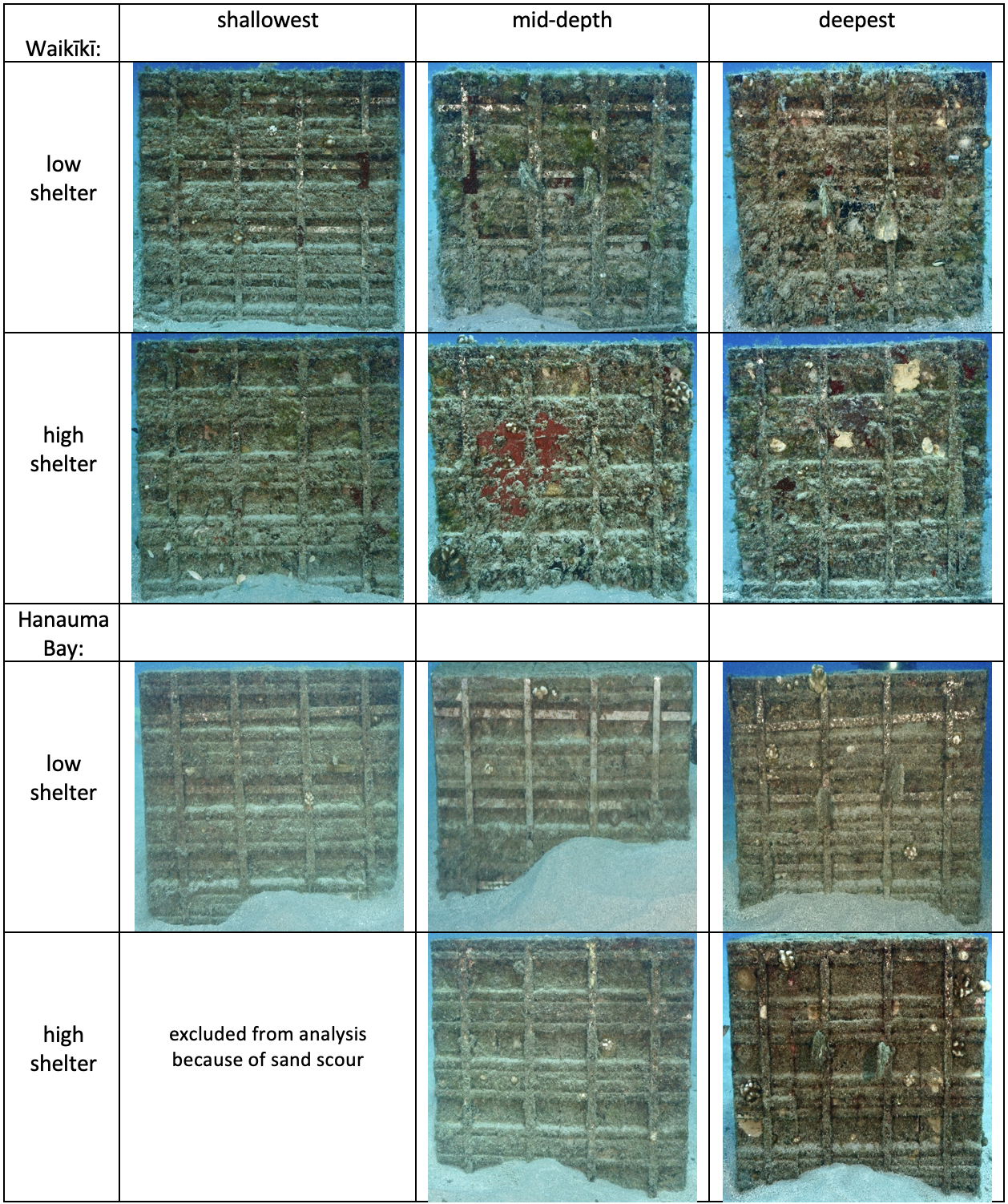

Supplement: Supplemental Information 3 — Photos near the end of the experiment of concrete modules at the relatively degraded reef of Waikı¯kı¯ (9/26/2019) and the relatively healthy reef of Hanauma Bay (9/24/2019). Turf, macroalgae, and encrusting sessile benthos (e.g., sponges, ascidians, and bryozoans), all of which displace corals, were much more prevalent at Waikı¯kı¯, whereas larger coral colonies were more prevalent at Hanauma Bay. [file peerj-14-20891-s003.png]

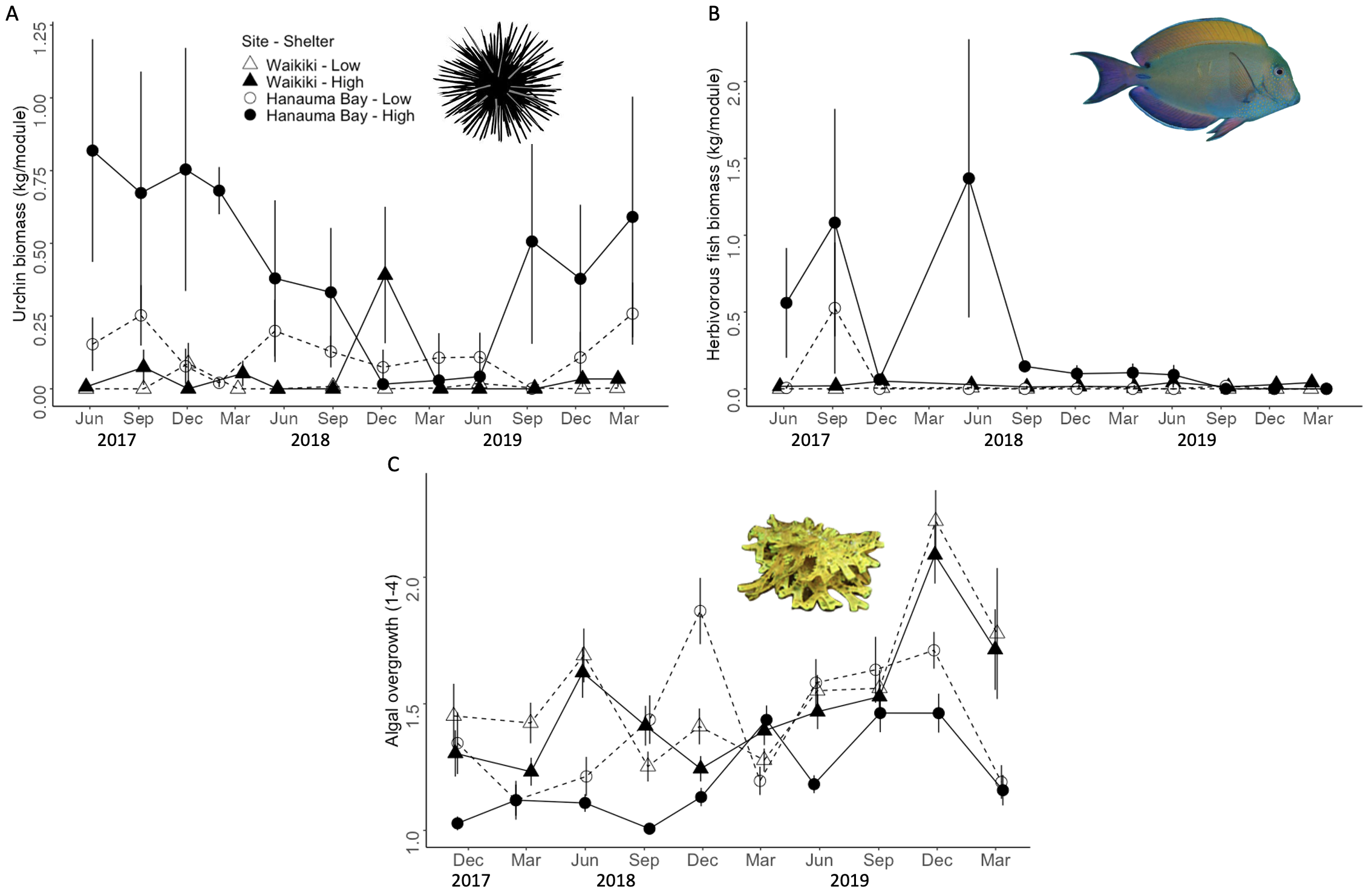

Supplement: Supplemental Information 4 — Time series plots (mean ±standard error) for (A) urchin biomass, (B) herbivorous fish biomass, and (C) algal overgrowth (n = 3 modules, except for one high-shelter module excluded from Hanauma Bay). [file peerj-14-20891-s004.png]

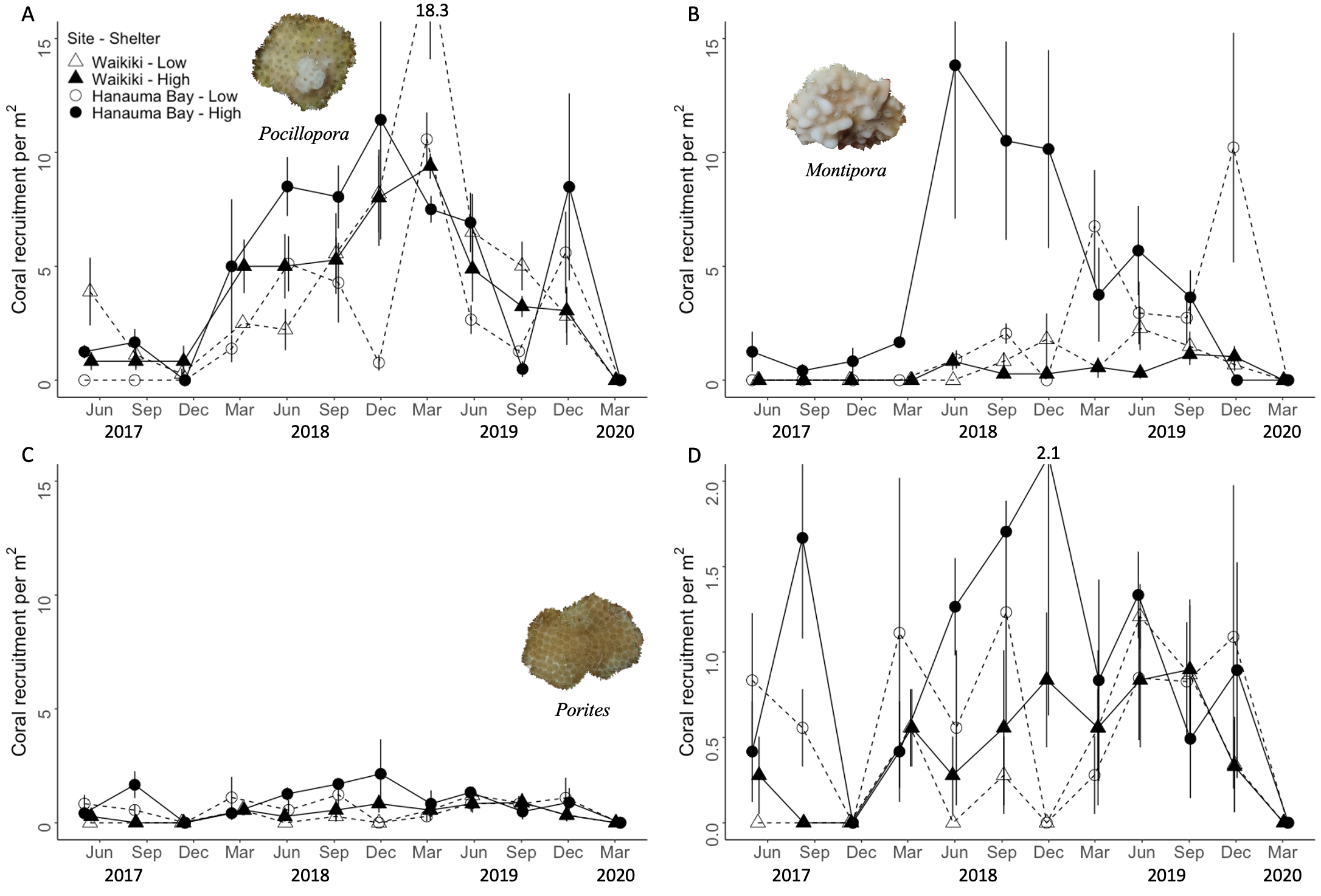

Supplement: Supplemental Information 5 — Time series plots for recruitment (mean ±standard error, n = 3 modules, except for one high-shelter module excluded from Hanauma Bay) for recruitment of (A) Pocillopora, (B) Montipora, (C) Porites, and (D) Porites re-scaled for easier interpretation. [file peerj-14-20891-s005.png]

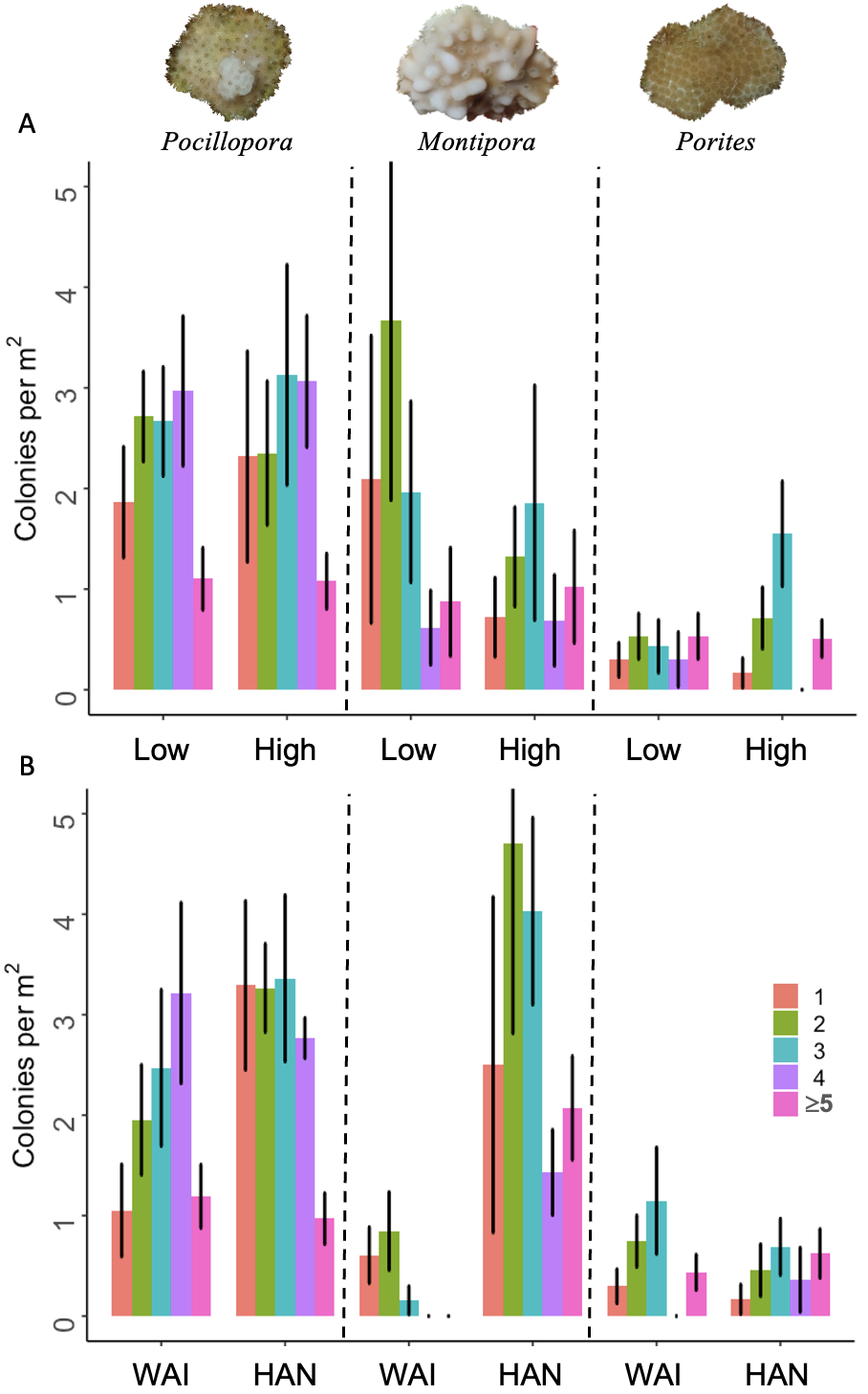

Supplement: Supplemental Information 6 — Coral colony abundance per meter squared of substrate at the last coral census during the study period (mean ±standard error, n = 3 modules, except for one high-shelter module excluded from Hanauma Bay). [file peerj-14-20891-s006.png]
