# Supplementary material for: Coral restoration: roles of shelter for herbivores and reef state in early recruitment success
Source: PeerJ. 2026 Apr 7;14:e20891. doi: 10.7717/peerj.20891 (PMC13068014; doi:10.7717/peerj.20891)
Supplement: Supplemental Information 7 — Summary table of R model functions used and equations for each hypothesis. Scale indicates whether response variables are on the module (e.g., recruitment as recruits per m2 of substrate of the entire module) or coral colony scale. Random effects for module replicate (1—m), quarter nested in year (1—t), and coral colony identity (1—ID) to account for repeated measures. [file peerj-14-20891-s007.pdf]

Hypothesis	Model (R Package)	Response Variable	Scale	Equation (Response ~ Predictors)
Herbivore	lmer (lme4)	Algal overgrowth (AO)	Module	AO ~ UB + HFB + R + S + (R × S) + (1|m) + (1|t)
Herbivore	lmer (lme4)	Coral recruitment (CR)	Module	CR ~ UB + HFB + AO + R + S + (R × S) + (1|m) + (1|t)
Herbivore	glmmTMB (glmmTMB)	Coral survival (CS)	Module	CS ~ UB + HFB + AO + R + S + (R × S) + (1|m) + (1|t)
Herbivore	lmer (lme4)	Coral growth (CG)	Module	CG ~ UB + HFB + AO + R + S + (R × S) + (1|m) + (1|t)
Reefscape/Shelter (R/S)	lmer (lme4)	Urchin biomass (UB)	Module	UB ~ R + S + (R × S) + (1|m) + (1|t)
Reefscape/Shelter (R/S)	lmer (lme4)	Herbivorous fish biomass (HFB)	Module	HB ~ R + S + (R × S) + (1|m) + (1|t)
Reefscape/Shelter (R/S)	lmer (lme4)	Algal overgrowth (AO)	Colony	AO ~ R + S + (R × S) + (1|ID) + (1|m) + (1|t)
Reefscape/Shelter (R/S)	lmer (lme4)	Coral recruitment (CR)	Module	CR ~ R + S + (R × S) + (1|m) + (1|t)
Reefscape/Shelter (R/S)	glmmTMB (glmmTMB)	Coral survival (CS)	Module	CS ~ R + S + (R × S) + (1|m) + (1|t)
Reefscape/Shelter (R/S)	lmer (lme4)	Coral growth (CG)	Colony	CG ~ R + S + (R × S) + (1|ID) + (1|m) + (1|t)
