# Supplementary material for: Coral restoration: roles of shelter for herbivores and reef state in early recruitment success
Source: PeerJ. 2026 Apr 7;14:e20891. doi: 10.7717/peerj.20891 (PMC13068014; doi:10.7717/peerj.20891)
Supplement: Supplemental Information 8 — Herbivorous fishes and urchins observed on experimental modules (6 per site), comparable natural patch reefs (6 per site), and continuous reef transects (n = 5) during herbivore surveys at both Waikı¯kı¯ and Hanauma Bay. “X” denotes that at least one individual of that species was observed over 4 yr. “X*” denotes that at least one new recruit fish (four cm TL) of that species was observed. [file peerj-14-20891-s008.pdf]

**Species**	**Waikīkī**			**Hanauma Bay**		
**Herbivorous fishes**	**Modules**	**Patch reefs**	**Continuous reef**	**Modules**	**Patch reefs**	**Continuous reef**
Acanthurus blochii				X		
Acanthurus dussumieri		X		X	X	
Acanthurus leucopareius				X		
Acanthurus nigrofuscus	X	X*	X*	X	X	X*
Acanthurus nigroris	X					
Acanthurus olivaceus	X	X*	X		X*	X
Acanthurus triostegus	X*				X	
Acanthurus xanthopterus				X	X	
Calotomus carolinus	X*	X*	X		X	
Chlorurus spilurus				X*	X*	X
Ctenochaetus strigosus		X	X*	X	X*	X*
Naso brevirostris	X					
Naso lituratus		X	X		X	X
Naso unicornis	X*	X				
Scarus psittacus		X	X		X	
Scarus rubroviolaceus	X*			X*		
Zebrasoma flavescens	X	X*	X*		X*	X*
**Urchins**	**Modules**	**Patch reefs**	**Continuous reef**	**Modules**	**Patch reefs**	**Continuous reef**
Chondrocidaris gigantea	X		X	X	X	X
Diadema paucispinum		X	X	X		X
Diadema savignyi		X	X			
Echinostrephus aciculatus			X		X	
Echinothrix calamaris	X	X	X	X	X	X
Echinothrix diadema		X		X	X	
Echinometra mathaei	X	X	X	X	X	X
Echinometra oblonga						X
Eucidaris metularia	X	X	X	X	X	X
Heterocentrotus mamillatus		X	X	X	X	X
Tripneustes gratilla	X		X	X	X	X
