# Supplementary material for: Coral restoration: roles of shelter for herbivores and reef state in early recruitment success
Source: PeerJ. 2026 Apr 7;14:e20891. doi: 10.7717/peerj.20891 (PMC13068014; doi:10.7717/peerj.20891)
Supplement: Supplemental Information 9 — Mean ±standard error percent cover for each benthic taxon and bare space on surveyed module sides (north and south walls combined) for Waikı¯kı¯-Low Shelter, Waikı¯kı¯ -High Shelter, Hanauma Bay-Low Shelter, and Hanauma Bay-High Shelter modules. Based on photographs taken at the beginning and end of the summer from 2017 to 2019 (TimeSteps: 1, 2, 5, 6, 9, 10). [file peerj-14-20891-s009.pdf]

Benthic Group	*Waikīkī-Low*	*Waikīkī-High*	*Hanauma Bay-Low*	*Hanauma Bay-High*
**Algae**:				
Turf	81.68 ± 1.65 %	82.89 ± 1.31 %	84.41 ± 1.91 %	76.41 ± 3.36 %
Macroalgae	7.47 ± 0.71 %	7.89 ± 0.92 %	6.68 ± 1 %	5.96 ± 0.99 %
Crustose coralline	0 ± 0 %	0.17 ± 0.09 %	0.33 ± 0.11 %	2.91 ± 0.66 %
**Invertebrates**:				
Ascidians/Bryozoans/Sponges	2.18 ± 0.56 %	4.67 ± 0.84 %	0 ± 0 %	0.06 ± 0.05 %
Hydroids	0.06 ± 0.05 %	0.28 ± 0.19 %	0 ± 0 %	0 ± 0 %
Worms	3.67 ± 1.47 %	0.56 ± 0.44 %	0 ± 0 %	0 ± 0 %
Bare Substrate	2.75 ± 0.49 %	1.39 ± 0.34 %	6.46 ± 1.31 %	10.55 ± 2.85 %
