# Supplementary material for: Coral restoration: roles of shelter for herbivores and reef state in early recruitment success
Source: PeerJ. 2026 Apr 7;14:e20891. doi: 10.7717/peerj.20891 (PMC13068014; doi:10.7717/peerj.20891)
Supplement: Supplemental Information 10 — Columns represent the five mm diameter size class at the beginning of a quarter while rows represent the size class at the end of the quarter. Cells contain mean probabilities of either growth (below the diagonal line), stasis (along the diagonal), or shrinkage (above the diagonal) for each possible size class transition with associated standard error. The third to last row shows cases where corals grew to greater than 20 mm in diameter. The second to last row (M) shows percent mortality during each transition, such that the sum of values in each column equals 1.0. The last row (n) represents the total number of observations for each given size class. [file peerj-14-20891-s010.pdf]

Low Shelter	Initial Size Class 1	2	3	4
End Quarter Size Class 1	0.22 ± 0.041	0.009 ± 0.005	0.007 ± 0.004	0
2	0.252 ± 0.038	0.229 ± 0.034	0.038 ± 0.011	0.02 ± 0.01
3	0.101 ± 0.028	0.355 ± 0.037	0.176 ± 0.03	0.032 ± 0.012
4	0.045 ± 0.017	0.184 ± 0.036	0.447 ± 0.046	0.288 ± 0.046
≥5	0	0.05 ± 0.021	0.165 ± 0.034	0.571 ± 0.047
M	0.381 ± 0.044	0.173 ± 0.031	0.169 ± 0.034	0.089 ± 0.021
n	296	288	263	225

High Shelter	Initial Size Class 1	2	3	4
End Quarter Size Class 1	0.237 ± 0.029	0.034 ± 0.011	0.009 ± 0.007	0
2	0.32 ± 0.038	0.251 ± 0.035	0.066 ± 0.018	0.039 ± 0.02
3	0.152 ± 0.042	0.338 ± 0.034	0.227 ± 0.037	0.063 ± 0.023
4	0.012 ± 0.008	0.193 ± 0.041	0.372 ± 0.037	0.222 ± 0.035
≥5	0	0.049 ± 0.024	0.207 ± 0.038	0.563 ± 0.045
M	0.279 ± 0.036	0.136 ± 0.031	0.119 ± 0.031	0.113 ± 0.03
n	339	303	244	208

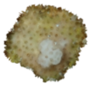

Pocillopora

Waikiki	Initial Size Class 1	2	3	4
End Quarter Size Class 1	0.212 ± 0.028	0.025 ± 0.008	0.011 ± 0.006	0
2	0.3 ± 0.03	0.191 ± 0.027	0.068 ± 0.015	0.023 ± 0.009
3	0.148 ± 0.036	0.33 ± 0.031	0.194 ± 0.025	0.054 ± 0.014
4	0.044 ± 0.015	0.196 ± 0.037	0.366 ± 0.031	0.268 ± 0.034
≥5	0	0.075 ± 0.026	0.186 ± 0.031	0.502 ± 0.035
M	0.297 ± 0.033	0.183 ± 0.03	0.175 ± 0.028	0.152 ± 0.025
n	384	409	373	326

Hanauma Bay	Initial Size Class 1	2	3	4
End Quarter Size Class 1	0.251 ± 0.045	0.013 ± 0.008	0.003 ± 0.003	0
2	0.268 ± 0.049	0.303 ± 0.042	0.027 ± 0.014	0.036 ± 0.023
3	0.099 ± 0.036	0.37 ± 0.042	0.21 ± 0.044	0.036 ± 0.023
4	0.008 ± 0.008	0.177 ± 0.039	0.472 ± 0.057	0.239 ± 0.052
≥5	0	0.015 ± 0.009	0.183 ± 0.043	0.66 ± 0.059
M	0.374 ± 0.05	0.121 ± 0.032	0.104 ± 0.038	0.028 ± 0.022
n	251	182	134	107
