# Supplementary material for: Coral restoration: roles of shelter for herbivores and reef state in early recruitment success
Source: PeerJ. 2026 Apr 7;14:e20891. doi: 10.7717/peerj.20891 (PMC13068014; doi:10.7717/peerj.20891)
Supplement: Supplemental Information 16 — Algal overgrowth was analyzed using the lmer function. σ2 and t00 represent the residual variance and random effect variance explained respectively. [file peerj-14-20891-s016.pdf]

	Algal overgrowth vs Herbivore biomass			
Predictors	*Estimate*	*SE*	*t value*	*p*
Urchin biomass (kg)	-0.11	0.13	-0.83	0.407
Herbivorous fish biomass (kg)	-0.04	0.12	-0.34	0.731
Site	-0.12	0.05	-2.29	**0.048**
Shelter	-0.06	0.05	-1.25	0.246
Site x Shelter	-0.12	0.07	-1.70	0.131
**Random Effects**				
σ^2	0.08			
τ_{00} module_urchin_and_fish_biomass_vs_algae	0.00			
τ_{00} Season:Year	0.02			
τ_{00} Year	0.00			
Observations	99			
Marginal R^2 / Conditional R^2	0.119 / 0.343			
