# Supplementary material for: Coral restoration: roles of shelter for herbivores and reef state in early recruitment success
Source: PeerJ. 2026 Apr 7;14:e20891. doi: 10.7717/peerj.20891 (PMC13068014; doi:10.7717/peerj.20891)
Supplement: Supplemental Information 17 — Recruitment was analyzed using the lmer function. Recruitment response variable was log(x + 1) transformed for all three genera. σ2 and t00 represent the residual variance and random effect variance explained respectively. [file peerj-14-20891-s017.pdf]

	PC Recruitment				MO Recruitment				PR Recruitment			
Predictors	*Estimate*	*SE*	*t value*	*p*	*Estimate*	*SE*	*t value*	*p*	*Estimate*	*SE*	*t value*	*p*
Urchin biomass (kg)	-0.21	0.23	-0.92	0.357	0.35	0.29	1.23	0.222	-0.09	0.16	-0.56	0.574
Herbivorous fish biomass (kg)	0.29	0.22	1.32	0.190	0.46	0.26	1.75	0.084	0.19	0.15	1.30	0.196
Algal overgrowth (1-4)	-0.81	0.19	-4.15	**<0.001**	-0.31	0.22	-1.43	0.155	-0.11	0.13	-0.84	0.401
**Random Effects**												
σ^2	0.31				0.42				0.13			
τ_{00}	0.00 module_urchin_fish_algae_recruit				0.13 module_urchin_fish_algae_recruit				0.03 module_urchin_fish_algae_recruit			
	0.10 Season:Year				0.00 Season:Year				0.01 Season:Year			
	0.82 Year				0.23 Year				0.05 Year			
Observations	99				99				99			
Marginal R^2 / Conditional R^2	0.063 / 0.761				0.050 / 0.492				0.021 / 0.407			
