# Supplementary material for: Coral restoration: roles of shelter for herbivores and reef state in early recruitment success
Source: PeerJ. 2026 Apr 7;14:e20891. doi: 10.7717/peerj.20891 (PMC13068014; doi:10.7717/peerj.20891)
Supplement: Supplemental Information 18 — Survival was analyzed using the glmmTMB function with a binomial distribution whereas growth was analyzed using the lmer function. σ2 and t00 represent the residual variance and random effect variance explained respectively. [file peerj-14-20891-s018.pdf]

	PC 1-5 Survival				PC 6-10 Survival				PC 11-15 Survival				PC 16-20 Survival			
Predictors	*Estimate*	*SE*	*t value*	*p*	*Estimate*	*SE*	*t value*	*p*	*Estimate*	*SE*	*t value*	*p*	*Estimate*	*SE*	*t value*	*p*
Urchin biomass (kg)	0.70	0.46	1.53	0.126	0.40	0.67	0.60	0.546	0.86	1.21	0.70	0.481	0.03	1.31	0.02	0.982
Herbivorous fish biomass (kg)	-0.04	0.29	-0.13	0.897	2.09	3.17	0.66	0.510	3.59	4.26	0.84	0.399	2.13	6.84	0.31	0.756
Algal overgrowth (1-4)	-0.75	0.34	-2.25	**0.024**	-0.50	0.51	-0.98	0.328	-0.58	0.45	-1.30	0.193	-1.51	0.76	-2.00	**0.046**
**Random Effects**																
σ^2	3.29				3.29				3.29				3.29			
τ_{00}	0.18 module_urchin_fish_algae_survival				0.13 module_urchin_fish_algae_survival				0.04 module_urchin_fish_algae_survival				0.84 module_urchin_fish_algae_survival			
	0.18 Season:Year				0.18 Season:Year				0.00 Season:Year				0.29 Season:Year			
	0.00 Year				0.00 Year				0.23 Year				0.00 Year			
Observations	89				91				91				95			
Marginal R^2 / Conditional R^2	0.127/0.504				0.321/0.562				0.538/0.666				0.248/0.669			

	PC 1-5 Growth				PC 6-10 Growth				PC 11-15 Growth				PC 16-20 Growth			
Predictors	*Estimate*	*SE*	*t value*	*p*	*Estimate*	*SE*	*t value*	*p*	*Estimate*	*SE*	*t value*	*p*	*Estimate*	*SE*	*t value*	*p*
Urchin biomass (kg)	0.02	0.10	0.16	0.870	0.18	0.18	1.01	0.316	-0.01	0.31	-0.03	0.974	-0.44	0.40	-1.11	0.270
Herbivorous fish biomass (kg)	-0.07	0.09	-0.78	0.438	0.01	0.16	0.08	0.936	0.02	0.24	0.09	0.926	0.37	2.32	0.16	0.874
Algal overgrowth (1-4)	0.00	0.08	0.06	0.954	-0.28	0.15	-1.88	0.064	-0.34	0.23	-1.51	0.142	-0.00	0.31	-0.00	0.998
**Random Effects**																
σ^2	0.05				0.17				0.37				0.78			
τ_{00}	0.00 module_urchin_fish_algae_growth				0.01 module_urchin_fish_algae_growth				0.00 module_urchin_fish_algae_growth				0.02 module_urchin_fish_algae_growth			
	0.00 Season:Year				0.00 Season:Year				0.02 Season:Year				0.00 Season:Year			
	0.10 Year				0.21 Year				0.05 Year				0.57 Year			
Observations	81				87				83				89			
Marginal R^2 / Conditional R^2	0.003/0.666				0.033/0.582				0.029/0.195				0.009/0.436			
