# Supplementary material for: Coral restoration: roles of shelter for herbivores and reef state in early recruitment success
Source: PeerJ. 2026 Apr 7;14:e20891. doi: 10.7717/peerj.20891 (PMC13068014; doi:10.7717/peerj.20891)
Supplement: Supplemental Information 19 — Survival was analyzed using the glmmTMB function with a binomial distribution whereas growth was analyzed using the lmer function. σ2 and t00 represent the residual variance and random effect variance explained respectively. [file peerj-14-20891-s019.pdf]

	MO 1-5 Survival				MO 6-10 Survival				MO 11-15 Survival				MO 16-20 Survival			
Predictors	*Estimate*	*SE*	*t value*	*p*	*Estimate*	*SE*	*t value*	*p*	*Estimate*	*SE*	*t value*	*p*	*Estimate*	*SE*	*t value*	*p*
Urchin biomass (kg)	1.86	1.16	1.60	0.110	-0.02	0.69	-0.03	0.977	-0.34	1.54	-0.22	0.825	-0.47	1.22	-0.38	0.495
Herbivorous fish biomass (kg)	7.50	4.69	1.60	0.110	2.39	2.83	0.85	0.397	16.77	7.10	2.36	**0.018**	253.86	742.80	0.34	0.458
Algal overgrowth (1-4)	0.56	0.83	0.67	0.502	-0.09	0.82	-0.11	0.911	-1.53	1.59	-0.96	0.336	-4.15	1.66	-2.50	**0.050**
**Random Effects**																
σ^2	3.29				3.29				3.29				3.29			
τ_{00}	0.85 $_{module\_urchin\_fish\_algae\_survival}$				0.00 $_{module\_urchin\_fish\_algae\_survival}$				2.42 $_{module\_urchin\_fish\_algae\_survival}$				0.00 $_{module\_urchin\_fish\_algae\_survival}$			
	0.26 $_{Season:Year}$				0.00 $_{Season:Year}$				0.00 $_{Season:Year}$				0.00 $_{Season:Year}$			
	1.01 $_{Year}$				0.00 $_{Year}$				1.56 $_{Year}$				0.00 $_{Year}$			
Observations	57				51				38				27			
Marginal R^2 / Conditional R^2	0.709/0.926				0.487/0.487				0.232/0.819				0.995/0.995			

	MO 1-5 Growth				MO 6-10 Growth				MO 11-15 Growth				MO 16-20 Growth			
Predictors	*Estimate*	*SE*	*t value*	*p*	*Estimate*	*SE*	*t value*	*p*	*Estimate*	*SE*	*t value*	*p*	*Estimate*	*SE*	*t value*	*p*
Urchin biomass (kg)	0.04	0.04	0.81	0.422	0.15	0.11	1.38	0.174	0.42	0.25	1.67	0.107	0.21	0.33	0.65	0.549
Herbivorous fish biomass (kg)	-0.02	0.03	-0.70	0.486	-0.05	0.09	-0.58	0.567	-1.14	1.38	-0.82	0.417	6.76	1.89	3.58	**0.006**
Algal overgrowth (1-4)	-0.06	0.04	-1.43	0.159	-0.21	0.11	-1.95	0.058	-0.24	0.25	-0.98	0.337	0.14	0.33	0.42	0.696
**Random Effects**																
σ^2	0.01				0.05				0.20				0.07			
τ_{00}	0.00 $_{module\_urchin\_fish\_algae\_growth}$				0.01 $_{module\_urchin\_fish\_algae\_growth}$				0.00 $_{module\_urchin\_fish\_algae\_growth}$				0.42 $_{module\_urchin\_fish\_algae\_growth}$			
	0.00 $_{Season:Year}$				0.00 $_{Season:Year}$				0.00 $_{Season:Year}$				1.00 $_{Season:Year}$			
	0.00 $_{Year}$				0.00 $_{Year}$				0.08 $_{Year}$				0.00 $_{Year}$			
Observations	48				47				34				23			
Marginal R^2 / Conditional R^2	0.062/0.330				0.136/0.230				0.086/0.356				0.121/0.960			
