# Supplementary material for: Coral restoration: roles of shelter for herbivores and reef state in early recruitment success
Source: PeerJ. 2026 Apr 7;14:e20891. doi: 10.7717/peerj.20891 (PMC13068014; doi:10.7717/peerj.20891)
Supplement: Supplemental Information 20 — Survival was analyzed using the glmmTMB function with a binomial distribution whereas growth was analyzed using the lmer function. σ2 and t00 represent the residual variance and random effect variance explained respectively. [file peerj-14-20891-s020.pdf]

	PR 1-5 Survival				PR 6-10 Survival				PR 11-15 Survival				PR 16-20 Survival			
Predictors	*Estimate*	*SE*	*t value*	*p*	*Estimate*	*SE*	*t value*	*p*	*Estimate*	*SE*	*t value*	*p*	*Estimate*	*SE*	*t value*	*p*
Urchin biomass (kg)	0.56	1.35	0.42	0.675	-0.94	0.96	-0.98	0.328	35.48	45.80	0.77	0.439	1.43	2.25	0.63	0.526
Herbivorous fish biomass (kg)	15.68	6.66	2.36	**0.018**	24.62	12.41	1.98	**0.047**	13.97	13.42	1.04	0.298	8.33	7.43	1.12	0.262
Algal overgrowth (1-4)	0.86	0.93	0.93	0.354	0.17	0.90	0.18	0.854	0.72	0.97	0.74	0.458	1.48	1.39	1.06	0.289
**Random Effects**																
σ^2	3.29				3.29				3.29				3.29			
τ_{00}	0.37 $_{module\_urchin\_fish\_algae\_survival}$				0.00 $_{module\_urchin\_fish\_algae\_survival}$				0.00 $_{module\_urchin\_fish\_algae\_survival}$				0.00 $_{module\_urchin\_fish\_algae\_survival}$			
	0.00 $_{Season:Year}$				0.00 $_{Season:Year}$				0.04 $_{Season:Year}$				0.15 $_{Season:Year}$			
	0.29 $_{Year}$				0.00 $_{Year}$				0.00 $_{Year}$				0.09 $_{Year}$			
Observations	50				59				58				53			
Marginal R^2 / Conditional R^2	0.928/0.946				0.976/0.976				0.984/0.984				0.816/0.837			

	PR 1-5 Growth				PR 6-10 Growth				PR 11-15 Growth				PR 16-20 Growth			
Predictors	*Estimate*	*SE*	*t value*	*p*	*Estimate*	*SE*	*t value*	*p*	*Estimate*	*SE*	*t value*	*p*	*Estimate*	*SE*	*t value*	*p*
Urchin biomass (kg)	0.19	0.24	0.78	0.440	-0.18	0.30	-0.60	0.553	0.42	0.26	1.64	0.109	0.39	0.46	0.84	0.407
Herbivorous fish biomass (kg)	-0.01	0.16	-0.09	0.932	-0.20	0.22	-0.88	0.385	0.25	0.21	1.18	0.243	0.05	0.33	0.14	0.893
Algal overgrowth (1-4)	-0.22	0.19	-1.16	0.257	-0.22	0.25	-0.85	0.398	-0.08	0.23	-0.37	0.716	-0.52	0.44	-1.19	0.244
**Random Effects**																
σ^2	0.15				0.31				0.25				0.60			
τ_{00}	0.01 $_{module\_urchin\_fish\_algae\_growth}$				0.00 $_{module\_urchin\_fish\_algae\_growth}$				0.00 $_{module\_urchin\_fish\_algae\_growth}$				0.16 $_{module\_urchin\_fish\_algae\_growth}$			
	0.00 $_{Season:Year}$				0.02 $_{Season:Year}$				0.19 $_{Season:Year}$				0.00 $_{Season:Year}$			
	0.00 $_{Year}$				0.00 $_{Year}$				0.00 $_{Year}$				0.46 $_{Year}$			
Observations	36				49				52				42			
Marginal R^2 / Conditional R^2	0.057/0.129				0.028/0.084				0.054/0.462				0.045/0.533			
