# Supplementary material for: Coral restoration: roles of shelter for herbivores and reef state in early recruitment success
Source: PeerJ. 2026 Apr 7;14:e20891. doi: 10.7717/peerj.20891 (PMC13068014; doi:10.7717/peerj.20891)
Supplement: Supplemental Information 21 — Urchin biomass and herbivorous fish biomass were analyzed using the glmmTMB function with a log link tweedie distribution and algal overgrowth was analyzed using the clmm function for ordinal data. σ2 and t00 represent the residual variance and random effect variance explained respectively. [file peerj-14-20891-s021.pdf]

	**Urchin biomass (Kg)**				**Herbivorous fish biomass (Kg)**				**Algal overgrowth (1-4)**			
Predictors	*Estimate*	*SE*	*t value*	*p*	*Estimate*	*SE*	*t value*	*p*	*Estimate*	*SE*	*t value*	*p*
Site	1.71	0.30	4.91	**<0.001**	1.32	0.35	3.81	**<0.001**	-0.44	0.12	-3.77	**<0.001**
Shelter	1.06	0.35	3.05	**0.002**	1.66	0.32	5.16	**<0.001**	-0.26	0.12	-2.19	**0.029**
Site x Shelter	-0.16	0.46	-0.34	0.731	0.08	0.47	0.17	0.864	-0.31	0.16	-1.89	0.059
**Random Effects**												
σ^2	0.00				0.00				3.29			
τ_{00}	0.13 $_{module\_urchins}$				0.00 $_{module\_fish}$				0.16 $_{id\_code\_algae}$			
	0.00 $_{Season:Year}$				0.62 $_{Season:Year}$				0.05 $_{module\_algae}$			
	0.07 $_{Year}$				0.19 $_{Year}$				0.28 $_{Year:Season}$			
Observations	132				121				3668			
Marginal R^2 / Conditional R^2	0.532/0.588				0.473/0.662				0.045 / 0.171			
