# Supplementary material for: Coral restoration: roles of shelter for herbivores and reef state in early recruitment success
Source: PeerJ. 2026 Apr 7;14:e20891. doi: 10.7717/peerj.20891 (PMC13068014; doi:10.7717/peerj.20891)
Supplement: Supplemental Information 22 — Recruitment was analyzed using the lmer function. Recruitment response variable was log(x + 1) transformed for all three genera. σ2 and t00 represent the residual variance and random effect variance explained respectively. [file peerj-14-20891-s022.pdf]

	PC Recruitment				MO Recruitment				PR Recruitment			
Predictors	*Estimate*	*SE*	*t value*	*p*	*Estimate*	*SE*	*t value*	*p*	*Estimate*	*SE*	*t value*	*p*
Site	-0.14	0.07	-1.89	0.062	0.44	0.13	3.52	**0.010**	0.14	0.10	1.33	0.224
Shelter	0.16	0.07	2.19	**0.031**	0.15	0.13	1.21	0.267	0.09	0.10	0.83	0.432
Site x Shelter	0.27	0.10	2.61	**0.010**	0.30	0.18	1.68	0.137	0.06	0.15	0.41	0.694
**Random Effects**												
σ^2	0.34				0.35				0.13			
τ_{00}	0.12 $_{Season:Year}$				0.01 $_{Season:Year}$				0.01 $_{Season:Year}$			
	0.00 $_{module\_recruitment}$				0.05 $_{module\_recruitment}$				0.05 $_{module\_recruitment}$			
	0.61 $_{Year}$				0.18 $_{Year}$				0.03 $_{Year}$			
Observations	132				132				132			
Marginal R^2 / Conditional R^2	0.037/0.697				0.160 / 0.501				0.053 / 0.420			
