# Supplementary material for: Coral restoration: roles of shelter for herbivores and reef state in early recruitment success
Source: PeerJ. 2026 Apr 7;14:e20891. doi: 10.7717/peerj.20891 (PMC13068014; doi:10.7717/peerj.20891)
Supplement: Supplemental Information 23 — Survival was analyzed using the glmmTMB function with a binomial distribution whereas growth was analyzed using the lmer function. σ2 and t00 represent the residual variance and random effect variance explained respectively. [file peerj-14-20891-s023.pdf]

	**PC 1-5 Survival**				**PC 6-10 Survival**				**PC 11-15 Survival**				**PC 16-20 Survival**			
Predictors	*Estimate*	*SE*	*t value*	*p*	*Estimate*	*SE*	*t value*	*p*	*Estimate*	*SE*	*t value*	*p*	*Estimate*	*SE*	*t value*	*p*
Site	-0.26	0.16	-1.66	0.098	0.45	0.21	2.11	**0.035**	0.53	0.27	1.96	**0.050**	1.59	0.56	2.82	**0.005**
Shelter	0.40	0.16	2.49	**0.013**	0.18	0.21	0.84	0.403	0.41	0.27	1.50	0.133	-0.01	0.56	-0.03	0.979
Site x Shelter	0.10	0.22	0.45	0.655	0.28	0.30	0.94	0.349	0.29	0.38	0.77	0.442	0.04	0.80	0.05	0.958
**Random Effects**																
σ^2	3.29				3.29				3.29				3.29			
τ_{00}	0.05 module_survival				0.05 module_survival				0.06 module_survival				0.22 module_survival			
	0.19 Season:Year				0.29 Season:Year				0.00 Season:Year				0.02 Season:Year			
	0.00 Year				0.00 Year				0.20 Year				0.17 Year			
Observations	99				109				111				113			
Marginal R^2 / Conditional R^2	0.130/0.404				0.107/0.427				0.179/0.394				0.493/0.659			

	**PC 1-5 Growth**				**PC 6-10 Growth**				**PC 11-15 Growth**				**PC 16-20 Growth**			
Predictors	*Estimate*	*SE*	*t value*	*p*	*Estimate*	*SE*	*t value*	*p*	*Estimate*	*SE*	*t value*	*p*	*Estimate*	*SE*	*t value*	*p*
Site	-0.09	0.03	-2.83	**0.005**	-0.05	0.07	-0.74	0.739	0.04	0.09	0.52	0.603	0.10	0.12	0.83	0.408
Shelter	-0.05	0.03	-1.69	0.091	-0.09	0.07	-1.35	0.296	-0.03	0.09	-0.41	0.684	-0.01	0.12	-0.07	0.946
Site x Shelter	0.07	0.04	1.51	0.131	0.11	0.10	1.10	0.387	0.00	0.12	0.03	0.975	0.17	0.17	0.98	0.327
**Random Effects**																
σ^2	0.05				0.20				0.67				1.91			
τ_{00}	0.08 id_code				0.14 id_code				0.33 id_code				0.00 id_code			
	0.01 Season:Year				0.01 Season:Year				0.15 Season:Year				0.12 Season:Year			
	0.00 module_growth				0.01 module_growth				0.00 module_growth				0.00 module_growth			
	0.08 Year				0.28 Year				0.13 Year				0.55 Year			
Observations	377				450				405				353			
Marginal R^2 / Conditional R^2	0.032/0.774				0.016/0.694				0.001/0.476				0.004/0.262			
