# Supplementary material for: Coral restoration: roles of shelter for herbivores and reef state in early recruitment success
Source: PeerJ. 2026 Apr 7;14:e20891. doi: 10.7717/peerj.20891 (PMC13068014; doi:10.7717/peerj.20891)
Supplement: Supplemental Information 24 — Survival was analyzed using the glmmTMB function with a binomial distribution whereas growth was analyzed using the lmer function. σ2 and t00 represent the residual variance and random effect variance explained respectively. [file peerj-14-20891-s024.pdf]

	MO 1-5 Survival				MO 6-10 Survival				MO 11-15 Survival				MO 16-20 Survival			
Predictors	*Estimate*	*SE*	*t value*	*p*	*Estimate*	*SE*	*t value*	*p*	*Estimate*	*SE*	*t value*	*p*	*Estimate*	*SE*	*t value*	*p*
Site	0.46	0.43	1.07	0.286	0.33	0.38	0.89	0.374	-6.58	7064.90	-0.00	0.999	-7.38	30898.19	-0.00	1.000
Shelter	-0.06	0.44	-0.15	0.883	0.23	0.38	0.61	0.542	6.90	7064.90	0.00	0.999	8.48	30898.19	0.00	1.000
Site x Shelter	0.96	0.62	1.55	0.120	0.18	0.53	0.34	0.733	-10.34	9991.28	-0.00	0.999	-11.45	43696.64	-0.00	1.000
**Random Effects**																
σ^2	3.29				3.29				3.29				3.29			
τ_{00}	0.40 $_{module\_survival}$				0.00 $_{module\_survival}$				0.15 $_{module\_survival}$				0.00 $_{module\_survival}$			
	0.77 $_{Season:Year}$				0.00 $_{Season:Year}$				0.72 $_{Season:Year}$				0.00 $_{Season:Year}$			
	0.37 $_{Year}$				0.00 $_{Year}$				1.18 $_{Year}$				0.00 $_{Year}$			
Observations	59				53				39				28			
Marginal R^2 / Conditional R^2	0.129/0.717				0.104/0.104				0.917/0.970				0.977/0.977			

	MO 1-5 Growth				MO 6-10 Growth				MO 11-15 Growth				MO 16-20 Growth			
Predictors	*Estimate*	*SE*	*t value*	*p*	*Estimate*	*SE*	*t value*	*p*	*Estimate*	*SE*	*t value*	*p*	*Estimate*	*SE*	*t value*	*p*
Site	0.03	0.03	0.95	0.345	0.16	0.05	2.93	**0.004**	0.23	0.16	1.50	0.137	0.12	0.44	0.28	0.784
Shelter	0.01	0.03	0.37	0.714	0.03	0.06	0.59	0.557	-0.09	0.16	-0.57	0.567	-0.17	0.44	-0.37	0.711
Site x Shelter	-0.03	0.04	-0.86	0.392	-0.05	0.08	-0.69	0.489	-0.06	0.22	-0.25	0.804	-0.14	0.64	-0.22	0.824
**Random Effects**																
σ^2	0.02				0.08				0.42				0.66			
τ_{00}	0.00 $_{id\_code}$				0.02 $_{id\_code}$				0.00 $_{id\_code}$				0.03 $_{id\_code}$			
	0.00 $_{Season:Year}$				0.01 $_{Season:Year}$				0.00 $_{module\_growth}$				0.48 $_{Season:Year}$			
	0.00 $_{module\_growth}$				0.00 $_{module\_growth}$				0.03 $_{Season:Year}$				0.47 $_{module\_growth}$			
	0.00 $_{Year}$				0.02 $_{Year}$				0.00 $_{Year}$				0.00 $_{Year}$			
Observations	188				159				84				55			
Marginal R^2 / Conditional R^2	0.013/0.120				0.050/0.345				0.028/0.092				0.014/0.603			
