# Supplementary material for: Coral restoration: roles of shelter for herbivores and reef state in early recruitment success
Source: PeerJ. 2026 Apr 7;14:e20891. doi: 10.7717/peerj.20891 (PMC13068014; doi:10.7717/peerj.20891)
Supplement: Supplemental Information 25 — Survival was analyzed using the glmmTMB function with a binomial distribution whereas growth was analyzed using the lmer function. σ2 and t00 represent the residual variance and random effect variance explained respectively. [file peerj-14-20891-s025.pdf]

	PR 1-5 Survival				PR 6-10 Survival				PR 11-15 Survival				PR 16-20 Survival			
Predictors	*Estimate*	*SE*	*t value*	*p*	*Estimate*	*SE*	*t value*	*p*	*Estimate*	*SE*	*t value*	*p*	*Estimate*	*SE*	*t value*	*p*
Site	-0.02	0.38	-0.06	0.951	-0.01	0.39	-0.04	0.971	-0.06	0.44	-0.14	0.886	0.35	0.53	0.67	0.506
Shelter	0.35	0.38	0.92	0.359	0.85	0.39	2.17	**0.030**	0.87	0.44	1.96	**0.050**	0.57	0.53	1.08	0.281
Site x Shelter	1.73	0.53	3.23	**0.001**	0.03	0.54	0.05	0.963	0.24	0.61	0.38	0.701	0.77	0.76	1.02	0.308
**Random Effects**																
σ^2	3.29				3.29				3.29				3.29			
τ_{00}	0.00 module_survival				0.00 module_survival				0.00 module_survival				0.00 module_survival			
	0.09 Season:Year				0.00 Season:Year				0.44 Season:Year				0.03 Season:Year			
	0.07 Year				0.08 Year				0.00 Year				0.30 Year			
Observations	54				69				69				58			
Marginal R^2 / Conditional R^2	0.282/0.334				0.177/0.217				0.137/0.299				0.146/0.280			

	PR 1-5 Growth				PR 6-10 Growth				PR 11-15 Growth				PR 16-20 Growth			
Predictors	*Estimate*	*SE*	*t value*	*p*	*Estimate*	*SE*	*t value*	*p*	*Estimate*	*SE*	*t value*	*p*	*Estimate*	*SE*	*t value*	*p*
Site	0.11	0.09	1.30	0.199	-0.18	0.08	-2.33	**0.022**	-0.03	0.11	-0.29	0.773	0.15	0.24	0.60	0.163
Shelter	-0.20	0.09	-2.23	**0.031**	-0.05	0.08	-0.66	0.508	0.06	0.11	0.54	0.592	-0.14	0.24	-0.58	0.301
Site x Shelter	-0.07	0.12	-0.57	0.572	0.24	0.11	2.26	**0.026**	0.11	0.15	0.75	0.455	0.20	0.36	0.55	0.305
**Random Effects**																
σ^2	0.00				0.11				0.50				1.00			
τ_{00}	0.13 id_code				0.14 id_code				0.00 id_code				0.00 id_code			
	0.00 Season:Year				0.02 Season:Year				0.06 Season:Year				0.14 module_growth			
	0.00 module_growth				0.00 module_growth				0.00 module_growth				0.17 Season:Year			
	0.00 Year				0.02 Year				0.12 Year				0.08 Year			
Observations	55				114				93				86			
Marginal R^2 / Conditional R^2	0.127/0.973				0.078/0.651				0.008/0.271				0.019/0.295			
